# Single-cell subcellular protein localisation using novel ensembles of diverse deep architectures

**DOI:** 10.1038/s42003-023-04840-z

**Published:** 2023-05-05

**Authors:** Syed Sameed Husain, Eng-Jon Ong, Dmitry Minskiy, Mikel Bober-Irizar, Amaia Irizar, Miroslaw Bober

**Affiliations:** 1 CVSSP, University of Surrey, Guildford, GU27XH Surrey UK; 2 ForecomAI, London, W1W 5PF UK

**Keywords:** Machine learning, Cell biology

## Abstract

Unravelling protein distributions within individual cells is vital to understanding their function and state and indispensable to developing new treatments. Here we present the Hybrid subCellular Protein Localiser (HCPL), which learns from weakly labelled data to robustly localise single-cell subcellular protein patterns. It comprises innovative DNN architectures exploiting wavelet filters and learnt parametric activations that successfully tackle drastic cell variability. HCPL features correlation-based ensembling of novel architectures that boosts performance and aids generalisation. Large-scale data annotation is made feasible by our AI-trains-AI approach, which determines the visual integrity of cells and emphasises reliable labels for efficient training. In the Human Protein Atlas context, we demonstrate that HCPL is best performing in the single-cell classification of protein localisation patterns. To better understand the inner workings of HCPL and assess its biological relevance, we analyse the contributions of each system component and dissect the emergent features from which the localisation predictions are derived.

## Abstract (web summary)

The Hybrid subCellular Protein Localiser (HCPL) improves single-cell classification for protein localization, allowing for large-scale data annotation through deep-learning architecture.

## Introduction

Proteins play a vital role in most cellular processes crucial to our survival. Their intracellular locations provide important insights about cell functions and state^1^. The specific biological functions that proteins perform are closely tied to the subcellular compartments in which they are expressed. Therefore, the subcellular resolution is critical in determining functional information about proteins and understanding the regulation of individual cells. A highly promising direction in this field is automated analysis of immunofluorescence microscopy images to enable large-scale impactful discoveries. For example, image-based spatial analysis of proteomic cellular heterogeneity can uncover a valuable view of protein expression with subcellular resolution, aiding the identification of disease biomarkers and drug discovery^2–4^.

Single-cell analysis is key to the detection of rare cells in heterogenous populations, essential in the profiling of tumour biology and precision medicine^5,6^. It constitutes a core strategy of the LifeTime Initiative, a large-scale, long-term initiative to implement cell-based interceptive medicine in Europe^7^. Although machine learning (ML) has been used to describe the location of human proteins in microscope images giving summary information on an entire population of cells^8–10^, subcellular classification of proteins for individual cells is still an open research area with limited published work^11^ and limited publicly available high-quality data^12^.

**Fig. 1:**
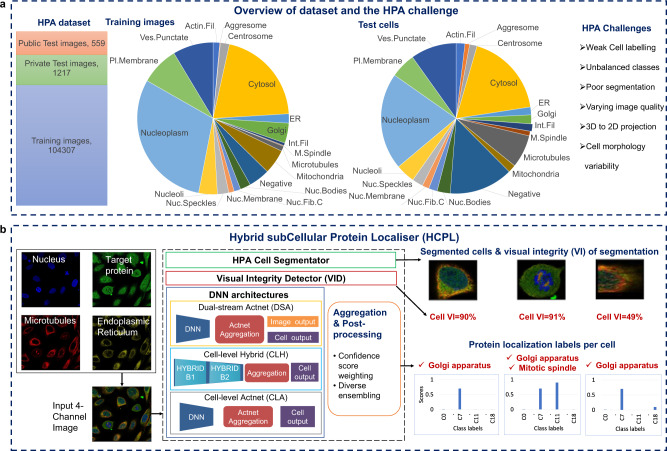
Overview of the HPA dataset and the proposed solution. **a** The HPA dataset is the largest collection of images depicting specific protein localisations at a subcellular level, acquired using immunofluorescence staining followed by confocal microscopy imaging. The training dataset consists of 104307 images and corresponding image-level labels. To evaluate the system’s performance, the test set comprises 1776 images of 41,597 single cells. The test set is divided into a public test set (559 images) and a private test set (1217 images). The pie charts illustrate the numerical proportion of images and cells per class in the training and test set. Developing ML models for protein localisation is challenging due to issues from weak labelling, prevalent multi-label classifications, 3D-2D projection ambiguities, and severe class imbalance. **b** Each HPA image is represented by four channels, the nucleus (blue), the protein of interest (green), microtubules (red), and the endoplasmic reticulum (yellow). Our HCPL system takes 4-channel images as input and outputs segmented cells, protein localisation labels with associated probabilities, and the visual integrity scores for each cell. Experimental evaluation shows that HCPL achieves the classification performance of 57.19% mAP in single-cell analysis.

To address this gap, we have developed a deep-learning-based system, the Hybrid subCellular Protein Localiser (HCPL), for robust protein localisation with subcellular resolution. Our system uses an ensemble of diverse deep architectures to generate precise annotations of protein localisation patterns in individual cells. We develop and validate our approach using the Human Protein Atlas (HPA)^13^, which is the largest public dataset (Fig. 1a) and forms an invaluable resource for studying cell biology (Methods). The HPA contains an extensive collection of four-channel images depicting specific protein localisations at a subcellular level, acquired using immunofluorescence staining followed by confocal microscopy imaging^14^. This resource is vital for understanding human cells and the complex molecular mechanisms underpinning their functions^15,16^, taking advantage of antibody-based multiplexed protein imaging methods^6,17^.

Aimed at individual cell analysis, the HCPL system successfully addresses several major challenges that this task raises. Compared with methods^9^ that can only provide predictions for a collection of cells (i.e., at the image level), the requirement to classify each cell individually is a far more difficult task. While single-cell localisation requires accurate predictions for each cell, on an image level it is sufficient to locate one relevant cell where the presence of a protein is most evident without classifying each of the remaining cells individually. From a ML perspective, this difficulty is further compounded by the frequent lack of accurate ground truth for training with typically only image-level labels available. For example, each HPA image comprises many cells jointly labelled with one set of labels, defined as the union of individual cell labels. Hence, the image-level labels are incorrect for some of the cells in an image. That is, in the same image of a genetically identical population, individual cells can exhibit different protein localisation patterns. This phenomenon is called weak labelling. Further difficulties arise from a dramatic variability in cells’ morphology, as well as from the use of different cell lines and inconsistent cell image quality caused by staining or segmentation failures. Finally, we must also contend with extremely imbalanced frequencies of the localisation classes, along with the multi-label setting where a single cell can take multiple labels. Details about HPA dataset class names and labels is presented in Supplementary Table 1.

Importantly, best image-level approaches^9^ were found unable to handle these challenges performing poorly on the task of cell-level protein localisation and only achieving circa 33.50% mAP. Recently presented unsupervised approach^10^ can learn latent space representations that loosely correspond to specific protein localisation patterns; however, this method cannot be used for the prediction of such patterns. Large-scale single-cell analysis and evaluation were made possible thanks to the efforts of Human Protein Atlas team who ran an open challenge on this particular topic called Human Protein Atlas - Single Cell Classification^12^. The results of this competition were analysed in ref. ^11^. However, in contrast to ref. ^11^ that reviews the best approaches in the competition, our work concentrates on the challenges that single-cell classification imposes and presents a systematic and in-depth solution. As a result, different parts of our system can be employed as stand-alone applications in the broader context (increasing the accuracy of existing labels and reducing the labour required for manual labelling tasks). In addition, the HCPL system produces superior results to those of^11^ and achieves the classification performance of 57.19% mAP in single-cell analysis.

To better understand our system’s operation and the benefits brought by its innovative components, we perform extensive testing, including a series of ablation studies. Furthermore, we benchmark our system against the leading solutions developed during the recent Kaggle competition (Human Protein Atlas - Single Cell Classification^12^). Finally, the work is concluded with an analysis that verifies the biological correctness and meaningfulness of the systems’ predictions.

We believe the HCPL system fills an important gap and is well-placed to contribute to our knowledge of spatial biology in health and disease, and its application to the development of therapeutics.

## Results

### HCPL - Hybrid subcellular protein localiser

Figure 1 presents an overview of the HPA dataset, the HPA challenge, and our HCPL solution. The HCPL system (Fig. 1b) receives multi-channel images, segments individual cells using the HPA Cell Segmentator (Methods), and analyses each cell in turn to estimate its visual integrity and the probabilities of proteins being present in specific subcellular compartments. HCPL combines several DNNs (Deep Neural Networks) to capture the biological variability and richness of patterns present in the HPA data, specifically:

- A multi-task Dual-stream Actnet (DSA) DNN, which learns to extract and fuse information from both images and individual cells. The DSA mitigates the vast amounts of false-positive cell predictions caused by the weak cell labelling used for training.
- A robust Cell-level Hybrid model (CLH), which combines learnt deep features with handcrafted features from a set of well-defined filters.
- A Cell-level Actnet (CLA), which employs learnable parametric activations to robustly aggregate discriminatory image features.

For system training, we propose an AI-trains-AI framework to improve the quality of the weak labels. It employs two techniques: (1) DNN-powered re-labelling, where labels are iteratively improved during the training, and (2) automatic adjustment of localisation confidence factors based on a cell’s estimated visual integrity, limiting the impact of cells with segmentation errors.

We develop an algorithm to select and optimally ensemble multiple classifiers to fully benefit from the diversity in expert opinions provided by different DNNs. Our HCPL system ensembles nine diverse architectures and achieves a protein localisation accuracy of 57.19% mAP, the highest among those evaluated. Execution time is below 1 second per cell image on a single thread CPU and NVIDIA-P100 GPU. It is essential to note that the test data is not available to anyone except competition organisers^11^. Our models are trained using only the training dataset without visibility of the test set and without external datasets. All cellular images presented in the manuscript have been obtained from the publicly available HPA dataset.

### DSA model for efficient cell-level and image-level information fusion

**Fig. 2:**
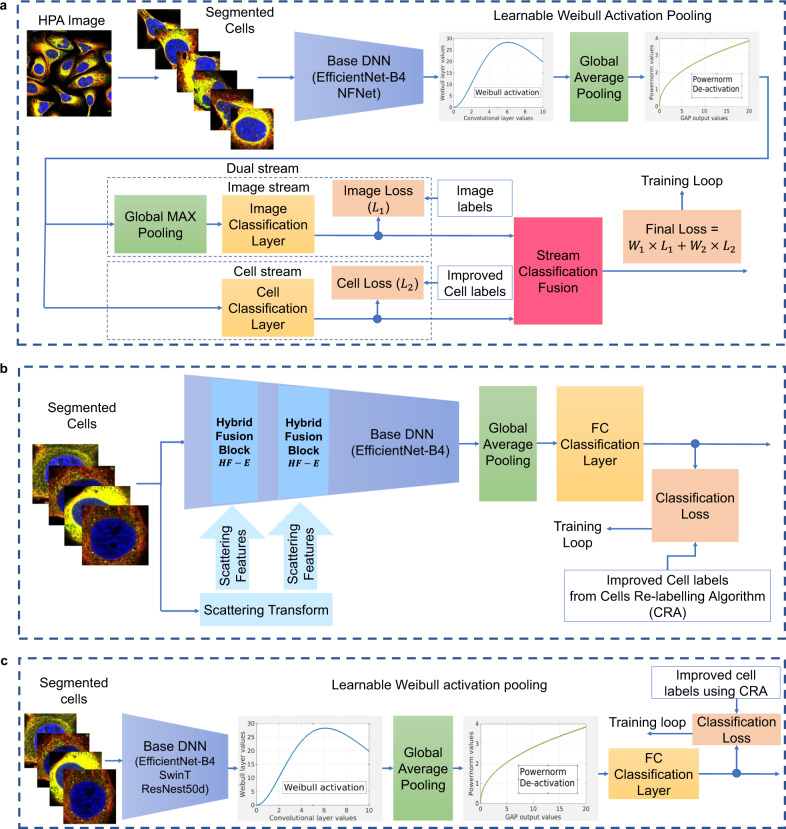
Key components of the HCPL system. **a** The DSA comprises an image stream that models the entire population of cells and a cell stream that classifies patterns in each cell in the image. **b** CLH is a single-stream inductive hybrid architecture that uses a scattering transform as a complimentary source of information about each cell. **c** CLA is a single-stream architecture that employs Weibull activation pooling to aggregate deep features before classification.

To maximise classification performance and increase robustness, our DSA model (Fig. 2a) jointly exploits local (cell-level) and global (image-level) visual cues. The DSA architecture comprises a baseline DNN (e.g., EfficientNet^18^) followed by the dual-stream network. From each image, cells are extracted and forwarded to the DNN component, producing deep convolutional features. The features are passed to a learnable Weibull activation Pooling^19^ to improve the discriminative power of the feature map (“Methods“). Specifically, the weak uninformative features are dampened, and a learnable proportion of strong informative features are equalised. The aggregated dense features are classified in two parallel streams—the image stream outputs predictions for the entire population of cells in an image, whereas the cell stream generates a set of predictions for each cell.

The DSA network is trained end-to-end using a weighted sum of Binary Cross-Entropy (Methods) losses from cell and image streams. At inference, for each cell, the probability of each class is computed as a product of relevant image and cell stream probabilities. For selected classes where image-level predictions are less reliable, the final probabilities given for such classes are the cell stream probabilities.

**Fig. 3:**
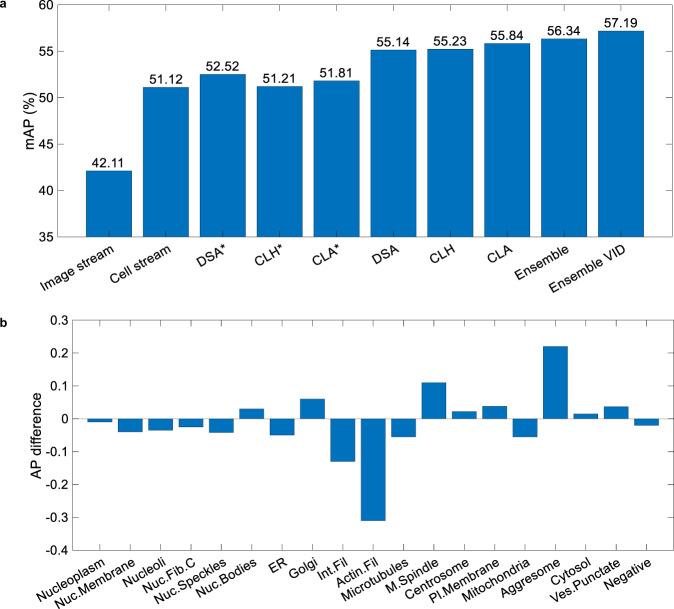
Performance details of selected elements of our system. **a** The classification performance of different modules of HCPL. The DSA*, CLH*, and CLA* modules are trained using weak image-level labels, whereas the DSA, CLH, and CLA modules are trained using improved labels obtained from the Cells Re-labelling Algorithm (CRA). The Ensemble VID and Ensemble systems represent the ensembling of DSA, CLA, and CLH models with and without the VID module, respectively. **b** Class-wise performance difference between cell-level Hybrid and cell-level Actnet. Positive values indicate better performance for CLH.

In Fig. 3a, experimental result show that the image stream individually achieves 42.11% mAP. This baseline performance comes from mapping image-level labels to all cells in that image, resulting in vast over labelling. The cell stream achieves a better 51.12% mAP, still relatively low because of the weak labels used in training. However, our dual-stream DSA architecture achieves 55.14%, a gain of (+4.02%) stemming from the intelligent fusion of both streams.

### Cell-level hybrid and actnet models for capturing cell variability

We develop two innovative cell-level architectures to comprehend the richness of patterns in the HPA. The first network, CLH, uses an inductive hybrid system (Fig. 2b) to fuse hand-crafted features extracted by a scattering transform with deep convolutional features. The scattering transform, formed by cascading wavelets, guarantees translation invariance and a linear response to deformations. The complementary nature of mathematically well-defined and data-adaptable filters yields a robust model. To ensure these properties are propagated into the DNN flow, two Hybrid Fusion blocks are inductively integrated into an EfficientNet architecture^20^.

The Hybrid fusion blocks have been designed to incorporate scattering features into the training process to enhance the data flow with representative pre-defined features. As a result, the CLH benefits from well-defined and informative hand-crafted features and an adaptable and flexible deep learning-based approach. Experimental results show that the Hybrid fusion blocks improve the performance by 1.58% mAP compared with the same DNN without fusion. The Hybrid-DNN module works on cell-level images and outputs convolutional features, which are aggregated and passed to the classification module to generate predictions.

The second network, CLA, uses parametric activations for adaptive and robust aggregation (Fig. 2c). At its core, a base DNN extracts features which are fed to a learnable activation layer^19^ comprising the Weibull function. The Weibull function amplifies responses corresponding to distinctive features of cells that are important for the classification tasks relative to the background. The transformed feature vectors are forwarded to a global average pooling layer and power-normalisation layer to generate a global vector. Experiments showed that Weibull pooling improves the mAP score by 2.82%, 2.53%, 2.11%, and 1.95% compared with the Global max pooling^21^, Global average pooling^22^, Region of interest pooling^23^, and Attention-based pooling^24^, respectively. The global vector is passed to the classification module to compute predictions.

CLH and CLA are trained using a weighted Binary Cross-Entropy (BCE) loss. The BCE weights for each class are calculated as the inverse of the class frequency in the training dataset. At inference, the class probabilities for each cell are computed similarly to the DSA, that is as a product of relevant DSA image stream and cell-level stream probabilities (CLA or CLH outputs), conditioned on the reliability of the image stream.

In Fig. 3b, we compute the class-wise Average Precision (AP) difference between CLH and CLA. It can be observed that the CLH better represents rare classes (0.22% and 0.11% AP gain over CLA on Aggresome and Mitotic spindle). The main gain comes from the handcrafted wavelet filters at the base of the Hybrid Fusion Block, which helps the network generalise with fewer training examples. Conversely, the CLA’s ability to select the most discriminative features helped it perform better on the visually similar Intermediate filaments (+0.13%) and Actin filaments (+0.31%).

### Estimation of cell visual integrity with VID

**Fig. 4:**
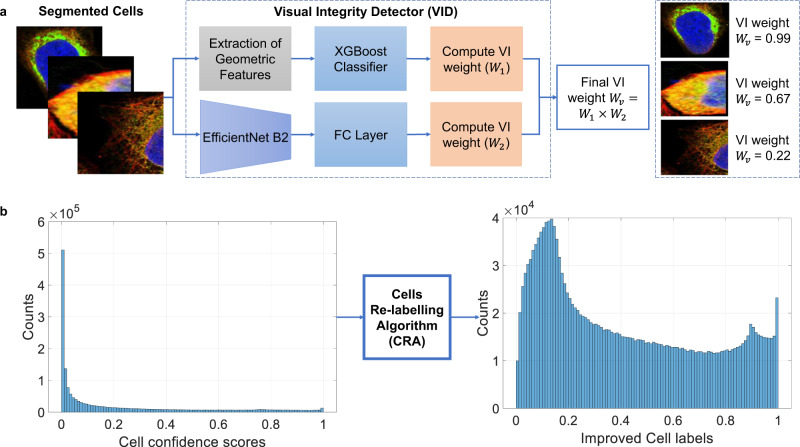
Functional details of selected elements of our system. **a** The VID system consists of two modules: one that extracts geometric features from a cell and employs an XGBoost classifier to evaluate its structural integrity, and a DNN to predict the ratio of the total cell body being captured. The final visual integrity weight *W*_*v*_ is computed by multiplying the weights *W*_1_ and *W*_2_, obtained from these two modules. **b** The figure on the left shows the histogram of confidence scores assigned to labels based on the ground-truth data. Approximately 60% of labels have scores below 0.1 indicating two issues: (1) DNNs are trained using weak labels, (2) training of DNNs is complex due to segmentation faults and cell variability. The CRA transforms the scores using a power-normalisation operation to generate a new set of probabilities *K*. The probabilities *K* then replace their associated labels, producing a new set of improved continuous valued cell labels. The histogram of improved cell labels is presented on the right.

Poor imaging or segmentation failures lead to errors in classification. Hence, an important feature of our system is a Visual Integrity Detector (VID) that is trained to detect cell capture errors and to adjust classification confidence accordingly. It is generally agreed that at least 50% of a cell has to be captured in an image for a meaningful prediction^12^. As presented in Fig. 4a, VID consists of two modules: (i) the first extracts hand-crafted geometric features from each cell and uses an XGBoost classifier^25^ to evaluate its structural integrity. (ii) An EfficientNet-B2^18^ network, which predicts the ratio of the total cell body being captured.

For training of the first VID module, we first compute the eight most representative features from each cell in the training dataset: bounding box height, width, aspect ratio, area, mask area, mask perimeter, the value of the largest dimension and a binary feature that is based on the pixel intensity and the ratio of blue and green to the total number of pixels. The VID dataset contains 10K cells hand-labelled as either ‘good’, i.e., most of a cell is visible or ‘bad’, i.e., a cell is severely occluded or a substantial part not visible. Note, this dataset with an extended set of properties and reference segmented cells is made publicly available^26^. We then train the XGBoost classifier on cell features using a fivefold cross-validation strategy. At inference time, each cell is passed to the XGBoost classifier to output the probability of the cell being bad (*P*_*b*_). The VI weight *W*_1_ is computed as 1 − *P*_*b*_.

The second module consists of a base EfficientNet-B2 with a fully connected layer to output predictions for four classes. The data to train the DNN is generated by randomly cropping out some area on the cell’s border. The cells are classified into four classes based on the proportion of their cropped area to the original cell: less than 30% belongs to class 1, 30%–50% belongs to class 2, 50%–80% belongs to class 3, and 80%–100% belongs to class 4. The network is trained using cross-entropy loss. During inference, the network is applied to each cell, and the weight *W*_2_ is assigned a value based on the output class, with *W*_2_ = 0.1 for class 1, *W*_2_ = 0.5 for class 2, and *W*_2_ = 1 for class 3 or 4.

The total weight *W*_*v*_ of VID is computed as the product of *W*_1_ and *W*_2_. Finally, the class probability of each cell is multiplied by *W*_*v*_. The accuracy of the VID is 92.05% on 2K cell images of the VID dataset. In Fig. 3a, we can observe that the inclusion of the VID module mitigates false positive cell predictions and improves the system performance by 0.85% mAP.

### Improving label quality using an AI-trains-AI approach

We leverage the generalisation ability of our DSA model to learn from noisy data and assign confidence scores to ground-truth labels (image-level labels naively mapped to cells). A confidence score represents the probability that a ground-truth label is correct; hence it takes values in [0,1]. We use confidence scores to perform iterative training where subsequent models focus less on low-confidence cell labels while emphasising those with high confidence. This is achieved by our Cells Re-labelling Algorithm (CRA). It first computes the per-cell component probabilities using three DSAs trained on weak labels (bases EfficientNet-B4, NFNet-ECA^27^, and ResNest50d^28^). These three component probabilities are averaged across each label for each cell to obtain a new set of combined confidence factors.

In the second step, we re-evaluate the true-positive labels for each cell based on these combined confidence factors (Fig. 4b, left). The CRA transforms the factors using a power-normalisation operation (*c* ↦ *c*^*β*^, *c* is a probability) to generate a new set of probabilities that replace the original labels, yielding improved continuous-valued cell labels (Fig. 4b right). The CRA effectively re-evaluates the weak cell labels originally inherited from the image-level labels. Next, we retrain the DSA model using re-labelled cells.

Results in Fig. 3a show that the DSA retrained on improved labels achieved performance of 55.14% mAP compared with DSA trained on original weak labels (52.52% mAP). The above process is employed twice to refine label quality. Finally, the cell labels obtained after round two are used to train the CLA and CLH models, leading to improvements exceeding +4% mAP over the models trained on the original labels.

### Multi-stream information fusion

**Fig. 5:**
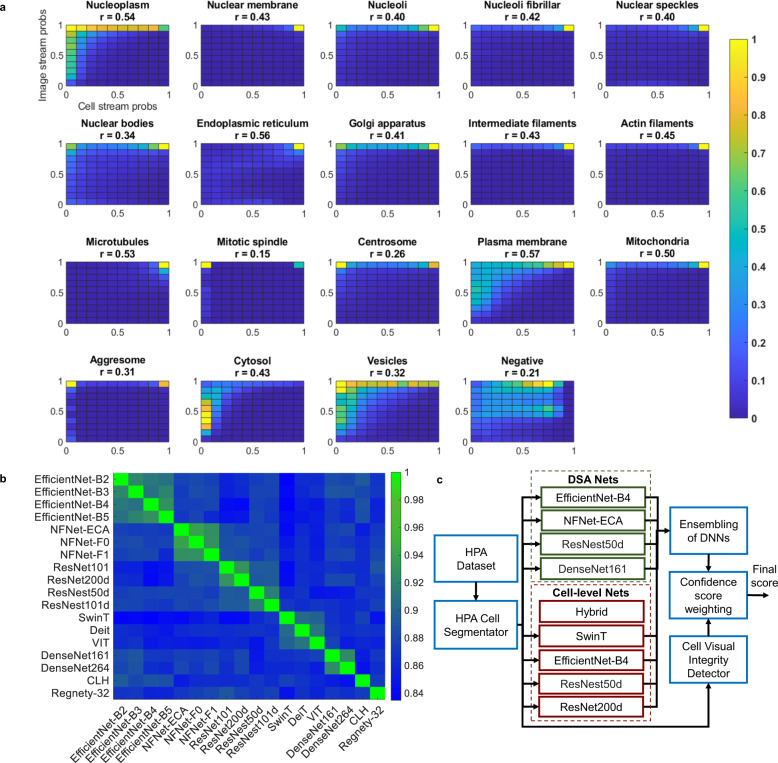
Correlation-based ensembling of diverse DNNs. **a** 2D histograms of image-level and cell-level predictions for all classes. Classes Mitotic spindle, Centrosome, Aggresome, and Vesicles have low correlation coefficients *r*. The values of *r* are displayed with the class names at the top of correlation plot. The image stream labels the majority of cells as having Aggresome, visible as a bright spots at the first row. However, we know that only around 30%–60% of cells in an image have Aggresome highlighted on the green channel. The cell-level stream is able to reject cells without Aggresome highlighted, causing a bright spot at the top-left corner of the histogram to appear. The same phenomenon can be seen for the class Mitotic spindle and Centrosome. **b** Correlation between probabilities generated by different DNNs. **c** HCPL inference framework employed to generate submissions for Kaggle private leaderboard.

We exploit the fusion of information extracted by selected classifiers working on image and cell levels to maximise the system’s performance. Figure 5a shows 2D histograms of image-level and cell-level predictions for all nineteen classes. Each histogram is computed using images with their corresponding label.

We note that certain classes (Mitotic spindle, Centrosome, Aggresome, and Vesicles) show disagreement in predictions, where a bright spot is present at the top-left corner. This shows cell-level labelling rejecting cells classified as positive by the image-level labelling. This phenomenon can be summarised by computing the correlation coefficient *r* between the image- and cell-level predictions. The aforementioned classes have very low correlation coefficient values, reflecting that these rare classes are prone to over-labelling by image classifiers.

Image and cell level fusion is performed as follows. For classes where the correlation is greater than a certain threshold *ρ*_*th*_ = 0.32, the localisation probabilities for a cell are obtained as per class products of image-level and cell-level predictions. For classes with low correlation, we assign the cell-level predictions to the final class probabilities. Please note that all cell-level networks are trained using improved cell-level labels.

### Robust protein localisation using diversity-based ensembling

**Fig. 6:**
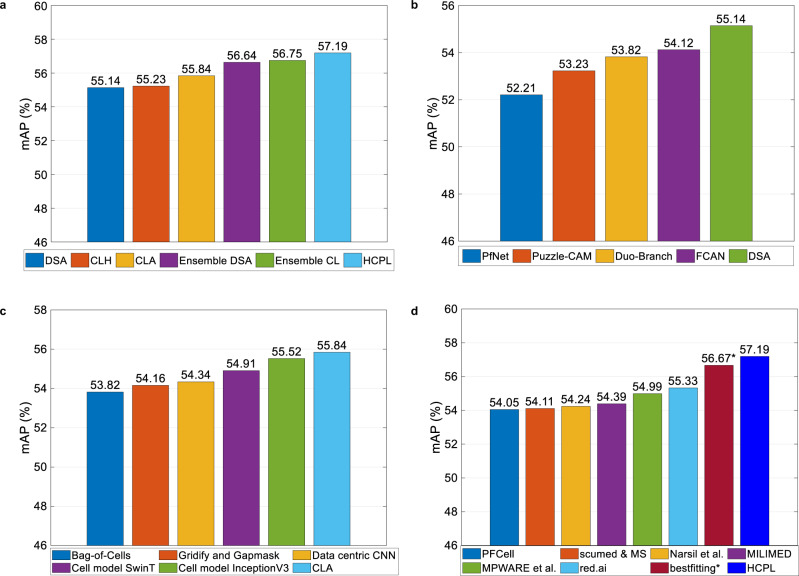
Evaluation of the HCPL system. **a** The impact of diversity-based ensembling of DNNs. **b** Comparison of our DSA with Kaggle multi-head models. **c** Comparison of our CLA with Kaggle cell-level models. **d** Comparison of our final system with existing work. Only bestfitting* system was trained using additional antibody-id information.

Experimental results of our models show that the maximum performance that a single model can achieve is 55.84% mAP (Fig. 6a). Classification accuracy can be substantially improved by optimal ensembling of DNNs (classifiers) outputs. However, meticulous selection of diverse classifiers is required to achieve this improvement. A set of classifiers are considered diverse if they perform well on different examples or classes.

The diversity inherent in the DNNs can be visualised with a correlation matrix of probabilities produced by each network (Fig. 5b). Here, we observe a block diagonal structure in the correlation matrix (green diagonal blocks), demonstrating that DNNs of similar architectures (EfficientNets, NFNets, ResNets, ResNests, DenseNets and Transformers) have strong correlations. The selection of the final network set (Fig. 5c) is accomplished by selecting the best-performing DNN, on the Kaggle public leaderboard (Methods), from each class of architectures.

The HCPL system, utilising nine diverse DNNs, is illustrated in Fig. 5c. The first phase is to extract individual cells from each image using HPA Cell Segmentator. Next, the cells are forwarded to the individual DSA and Cell-level networks to compute the predictions. The outputs from these diverse networks are hierarchically aggregated to compute the probabilities of all classes. Simultaneously, the cells are passed to the VID to compute the cell visual integrity weighting. Finally, the probabilities are multiplied by the visual integrity weights to generate the final vector of class probabilities.

### Quantifying HCPL performance and identifying its essential components

We conduct ablation studies to evaluate strengths of different models and the improvements brought by diversity-driven multi-DNN ensembling.

According to Fig. 6a, the individual DNNs are not able to improve beyond 55.84% mAP. The mAP is improved to 56.64% by ensembling four Dual-stream architectures with bases EfficientNet-B4^18^ (54.72%), NFNet-ECA^27^ (54.84%), ResNest50d^28^ (55.14%), and DenseNet161^29^ (54.67%). We can achieve a mAP of 56.75% by ensembling the predictions from CLAs with bases EfficientNet-B4 (55.61%), SwinT^30^ (54.95%), ResNet200d^31^ (55.84%), ResNest50d (55.22%) and CLH (55.23%). Importantly, the ensembling of all DSA and cell-level networks obtain a classification score of 57.19%.

### Benchmarking the HCPL system

We start our analysis with the evaluation of existing image-level systems. For this, we take the best-performing image-level classification DNN (bestfitting, DenseNet-based^9^) as a starting reference point. This model is fine-tuned on the HPA single-cell classification dataset using the original training configuration. The resulting performance is 33.50% mAP on single-cell classification, indicating that image-level classifiers are not directly applicable to the single-cell classification task. A more meaningful comparison can be obtained by evaluating the performance of HCPL against the methods employed in the Kaggle (HPA Single Cell Classification) competition^11,32^. The details about the different architectures are presented in Supplementary Note 1.

A limitation of this comparison is that existing solutions were designed during the four-month Kaggle competition, with feedback based on public leaderboard scores, while we developed HCPL after the competition. While we had no access to test data, submitting code after the competition showed scores on public and private leaderboards. For a fair comparison, we only used the public leaderboard to develop our algorithm and avoided any optimisation based on the private leaderboard. Once we finalised the architecture based on the public leaderboard, we reported the performance on the private leaderboard.

We first compare the performances of single models without ensembling. From Fig. 6b, we observe that the proposed DSA performs better than Kaggle multi-head models, including Fair Cell Activation Network (FCAN)^33^, Duo-branch^24^, modified Puzzle-CAM^34,35^, and PfNet^36^. This DSA improvement can be attributed to the use of Weibull activation pooling to aggregate deep features, correlation-dependent thresholding for the fusion of the two streams, along with training on improved labels from CRA.

AmonThang cell-level architectures, Fig. 6c demonstrates that our CLA achieves better classification performance (55.84%) than Cell model InceptionV3^33^, Cell model SwinT^33^, Data-centric CNN^37^, Gridify and Gapmask^38^, and Bag-of-Cells^39^. The advantage of CLA is associated with the use of learnable and parametric activation pooling to aggregate discriminatory features.

When ensemble-based architectures are considered (Fig. 6d), our HCPL system attains the highest classification accuracy of 57.19% mAP. The best currently published result (56.67%, bestfitting,^33^) was trained with antibody-id information which was scraped from the Human Protein Atlas website^40^ and re-linked with the competition dataset^41^.

The antibody-ids were excluded from the competition datasets by the organisers because their use in training presents three issues: (1) the model may use irrelevant hidden variables (for example, background noise) or focus on biologically meaningless features to improve the model performance^9,p. 1259]^, (2) the gains achieved might be due to the exploitation of batch effects and statistical correlations between the training and test sets^9,p. 1260]^, and (3) the model trained using antibody-id may generalise poorly to unseen cell lines, new antibody-ids, and unknown proteins.

In fact, competition organisers suggested limiting the set of information allowed for training to protein location labels only (as supplied with the datasets). This prevents models from exploiting spurious correlations between the predicted protein localisation labels and additional information such as antibody-ids^9,p. 1262]^, protein-ids^42^ and cell lines^43^, helping to ensure that the cell localisation predictions are unbiased and generalise well.

The evaluation results show that without using the antibody-id information, the performance of bestfitting is 55.54%. To achieve this outcome, we followed the code and algorithm description^11,33^ of the bestfitting solution, and retrained the models without incorporating the antibody-id information (Methods). Importantly, no other team, including our HCPL, used antibody-id information during model training. When a fair comparison is performed, i.e., antibody-id data is not employed, HCPL (57.19%) demonstrates a gain of +1.65% and +1.86% over the first (55.54%) and second (55.33%) Kaggle teams. The HCPL’s better performance is predominantly due to the combination of its component parts, which includes utilising DNN architectures with wavelet filters and Weibull activation pooling to effectively handle large cell variations, employing the CRA algorithm to produce enhanced cell labels, using the VID system to evaluate the visual quality of cells, and combining diverse deep architectures through correlation-based ensembling.

**Fig. 7:**
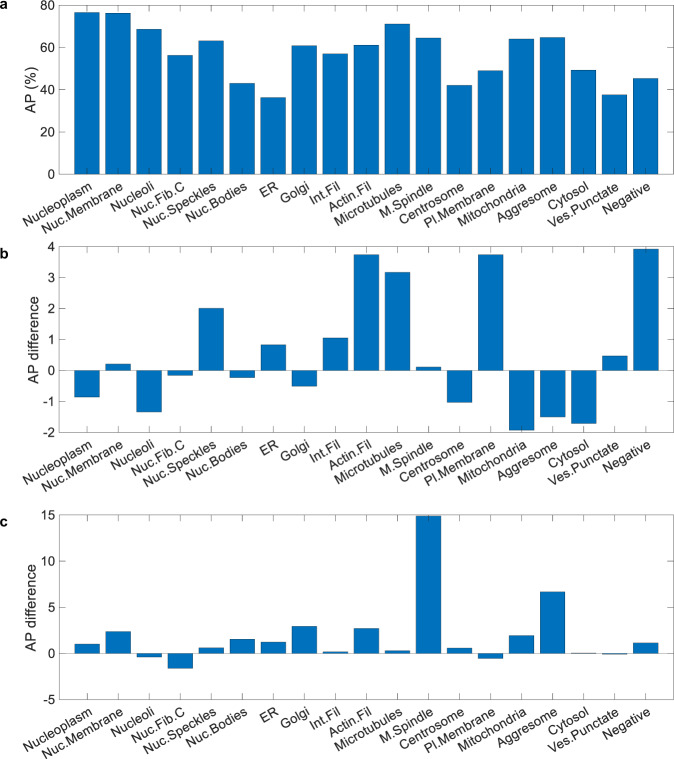
Class-wise performance of HCPL. **a** Analysis of class AP of HCPL shows a widespread, from 36.30% of Endoplasmic reticulum to 76.55% of Nucleoplasm. **b** Class-wise performance difference between HCPL and bestfitting (Kaggle top team). Positive values indicate better performance for HCPL. **c** Class-wise performance difference between HCPL and red.ai (Kaggle second team).

In Fig. 7a, we show the performance of our system in each of the 19 classes. The performance depends on several variables, such as the difficulty of recognising different localisation patterns, the number of training samples, and extreme visual variations. Classes with distinct visual patterns have high AP: Nucleoplasm 76.55%, Nuclear membrane 76.25%, and microtubules 71.10%. Despite a lower number of training samples, the AP is higher for Aggresome (64.75%) than Plasma membrane (49.02%) because Aggresome has visually distinguishable features, whereas Plasma membrane is often confused with Cytosol. The Endoplasmic reticulum has the lowest AP of 36.30%; it is also confused with Cytosol. Merged classes such as Centrosome (consisting of Centrosomes and Centriolar satellites) and Vesicles and Punctate cytosolic pattern (consisting of Vesicles, Peroxisomes, Endosomes, Lysosomes, Lipid droplets, and Cytoplasmic bodies) also have a very low AP of 42.10% and 37.60%, respectively. Despite the extreme rarity of the Mitotic spindle in training samples, our system achieves a good score of 64.50%.

In Fig. 7b, we demonstrate the class-wise AP difference between HCPL and Kaggle’s top team bestfitting. It is important to note that the HCPL was trained without using antibody-id information compared with bestfitting. Nevertheless, HCPL achieves superior AP in classes: Nuclear speckles (2.01%), Actin filaments (3.74%), Microtubules (3.17%), Plasma membrane (3.74%), and Negative (3.92%). On the other hand, the bestfitting method achieves better AP in classes: Nucleoli (1.34%), Mitochondria (1.93%), Aggresome (1.50%), and Cytosol (1.71%).

In Fig. 7c, we show the class-wise AP difference between HCPL and the existing best performer without antibody-id information, red.ai (Kaggle 2nd place). We can observe that HCPL compares favourably across the vast majority of classes. We also observe a higher performance advantage of our system in challenging classes due to the synergetic operation of the individual system’s components. For example, Cell-level Actnets help select the most discriminative features to distinguish Actin filaments (+2.71%) that are visually very similar to Intermediate filaments. Similarly, despite the small number of training samples, CLH benefits from pre-defined filters that help extract valuable features from the Mitotic spindle (+14.89%) and Aggresome (+6.67%). These features of HCPL combined with VID help achieve a considerable advantage in challenging classes over the red.ai system.

### The ability of HCPL to interpret biological information

We use the Grad-CAM method^44^ to identify the parts of an input image that impact the classification score. This highlights regions contributing features that support predictions of the target label, helping us understand whether localisation predictions are biologically meaningful (by comparing to the ground truth staining patterns). These attention regions will vary for each image and location label.

**Fig. 8:**
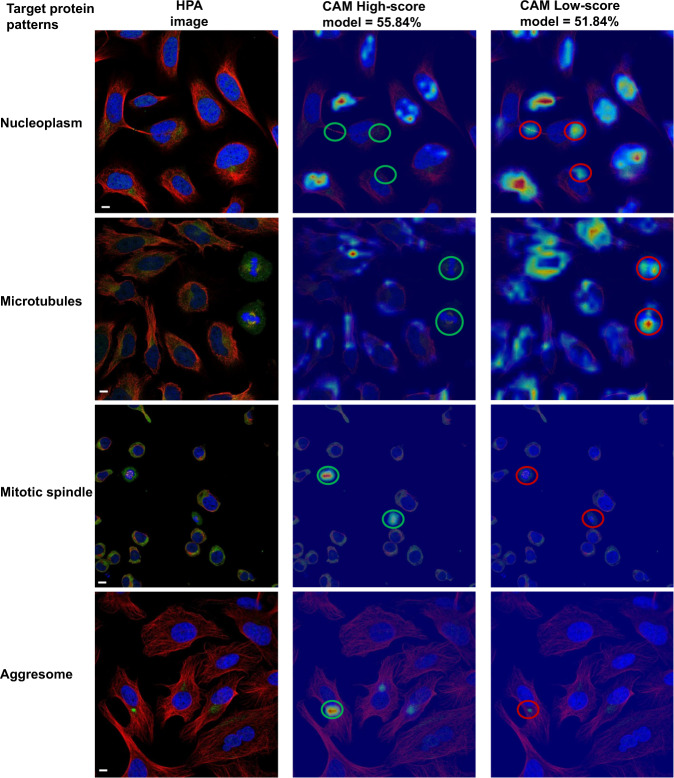
CAMs of the high-scoring CLA trained on CRA improved labels, and a low-scoring CLA trained on original weak labels. (1) The CAMs for the Nucleoplasm staining demonstrate biologically meaningless attention for the low-scoring model as shown by red circles. (2) The Microtubule staining overlaps well with the CAM for the high-scoring model. In contrast, the low-scoring model uses visual features of the Mitotic spindle class to assign a high probability for the Microtubule class, as indicated by red circles. (3) For the Mitotic spindle staining patterns, CAMs for the high-scoring CLA highlight relevant cellular regions indicated by green circles. (4) The red circle shows where the Aggresome staining pattern is not captured by the low-scoring DNN, whereas the green circle depicts the high-scoring model correctly localises it. Scale bars, 10 μm.

Figure 8 shows CAM regions for challenging patterns, such as Nucleoplasm, Microtubules, Mitotic spindle, and Aggresome. It compares the high-scoring CLA (trained using strong labels obtained from the CRA) and the low-scoring CLA (trained on weak cell labels). We can observe that the staining patterns for these difficult classes overlap well with the corresponding CAM attention regions of the high-scoring model, hence confirming that our high-scoring CLA focuses on biologically relevant cell regions.

### Deep features visualisation using UMAP

**Fig. 9:**
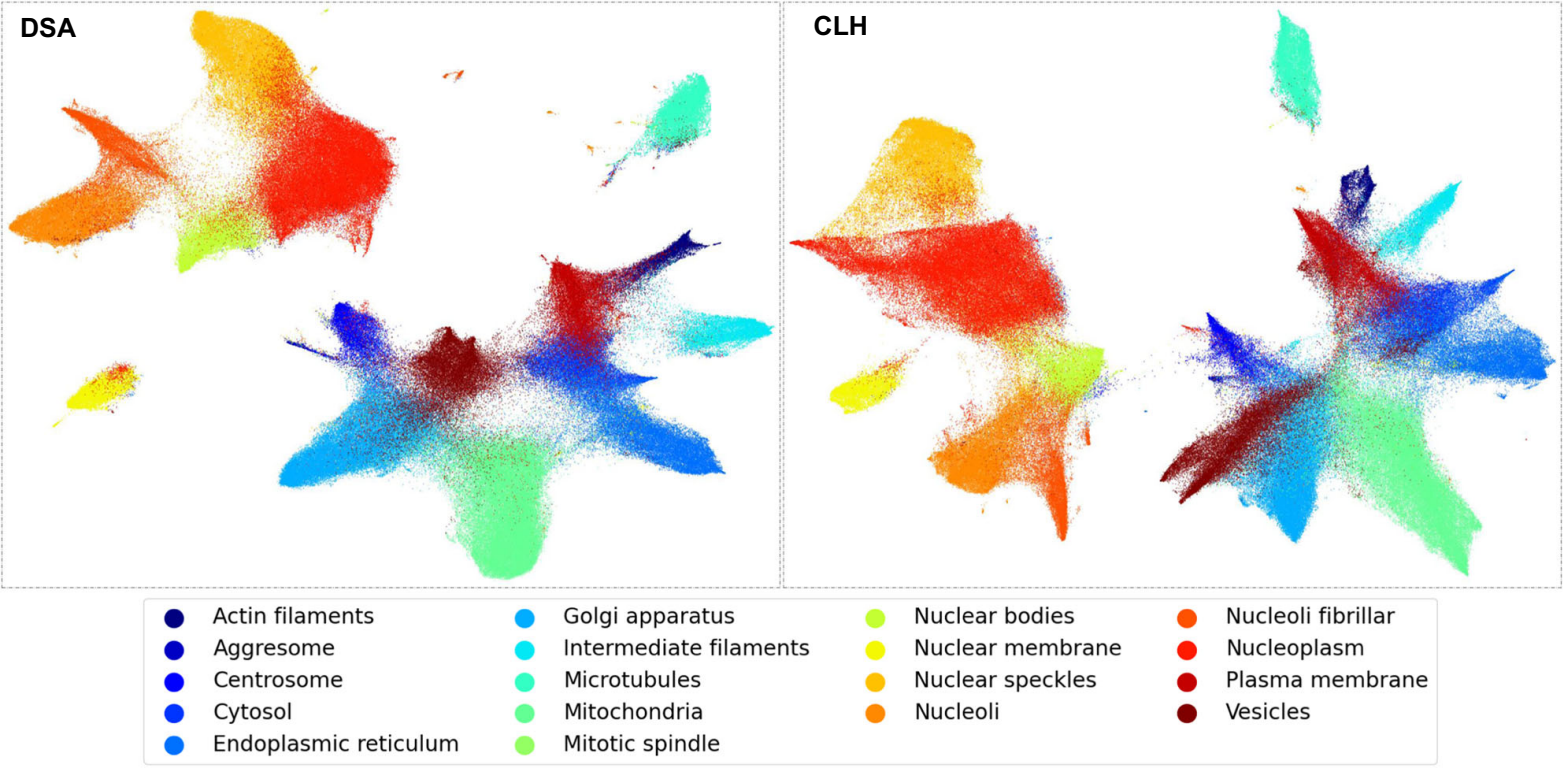
Visualisation of the deep features for DSA and CLH DNNs from the UMAP dimensionality reduction method. High-dimensional data is projected onto a 2D plane such that local structures in the original space are captured, whilst simultaneously retaining the global structure of the data. Different subcellular locations are effectively clustered by deep features in each model.

To investigate the ability of a DNN to distinguish subcellular structures, we visualise the high-dimensional feature vector extracted from the penultimate layer using uniform manifold approximation and projection for dimension reduction (UMAP)^45^. The visualisation results for two different DNNs (DSA and CLH) are presented in Fig. 9. Here, each point on the plot represents a single cell. Only cells with single labels are chosen, coloured by their respective label.

Firstly, we observe that the DNNs cluster the majority of cells from the same class together, which shows their underlying discriminative power. Secondly, we note the presence of two larger clusters at a global level: one for nuclear sub-compartments (Nucleoplasm, Nuclear membrane, Nucleoli, Nucleoli fibrillar centre, Nuclear speckles, and Nuclear bodies) and another for locations outside the nucleus (e.g., Cytosol, Mitochondria). Thirdly, instances of some classes are located within clusters belonging to other classes, which links to the relative decrease in performance (Fig. 7a), for example, Cytosol partially overlaps with Endoplasmic reticulum and Plasma membrane, Nuclear bodies with Nucleoplasm and Vesicles with Golgi apparatus. Lastly, the different amount of overlap observed in the clusters generated by DSA and CLH confirms the diversity and complementarity in the deep features.

## Discussion

Our core contributions include a multi-task DSA architecture for improved image and cell information fusion and two Cell-level architectures (CLA & CLH) to capture the wide variability between cells. Further, we introduce an AI-trains-AI approach with DNN-powered re-labelling and automatic cell visual integrity weighting and propose an effective strategy to optimally ensemble diverse DNNs. The evaluation shows that HCPL has better single-cell classification performance than existing approaches and is expected to generalise well to unseen cell lines and proteins. Further improvements could be achieved by obtaining more examples of rare localisation classes, accurate cell labels, and feedback from human experts.

HCPL narrows the performance gap between AI methods and human experts and provides a toolbox of methods to tackle the challenges of single-cell protein localisation successfully. This will help accelerate the characterisation of unknown proteins and our understanding of cellular function and biology to advance our knowledge of disease-related phenotypes and drug discovery.

## Methods

### HPA dataset

Our work uses the HPA dataset provided by the Human Protein Atlas - Single Cell Classification Kaggle challenge^12^. This consists of images from the freely accessible Human Protein Atlas project. In particular, images from the subcellular section of the HPA were used. A total of 104307 images (internal and external dataset) were made available for training purposes whilst an additional 1776 unseen images were retained by the challenge organisers for testing purposes, further split into 559 images for the public leaderboard and 1217 images for the private leaderboard. Each of the provided images contains multiple cells and consists of four channels, displayed in red, green, blue, and yellow. The task considered in this paper is to localise the protein of interest (green channel) in 18 possible subcellular organelles in each cell in an image. An additional negative class is added for negative staining and unspecific patterns.

A cell can have multiple labels (specifically, we work with 19 localisation classes with up to 6 protein locations per cell). The 19 labels and their names are shown in Fig. 1a. Our DNNs are trained on approximately 1.2 Million cell images segmented from HPA images using the HPA Cell Segmentator.

### Evaluation metric

To ensure a fair comparison of results, all experiments were evaluated by computing mean Average Precision (mAP)^46^. The mean value was calculated over the 19 segmentable classes (*C*) of the challenge with a mask-to-mask IoU > 0.6 as described below:

1 $${{{{{{{\bf{mAP}}}}}}}}=\frac{1}{C}\mathop{\sum }\limits_{i}^{C}Pre{c}_{i}$$

where *Prec*_*i*_ is Precision for class *i* which is calculated according to the two-stage method described in article^47^.

All mAP scores are reported based on the Kaggle private leaderboard.

### HPA cell segmentator (HCS)

The HCS (https://github.com/CellProfiling/HPA-Cell-Segmentation) segments input images into individual cell instances for multi-label classification. Since the procedure of the HCS software provided by organisers occupied 60% of the total permitted processing time (9 hours for the entire system’s inference), improving the efficiency of the segmentation algorithm is important. The algorithm consists of three main stages: (i) prediction of the nuclei; (ii) general cell prediction; and (iii) post-processing procedure. However, cell segmentation training data was not publicly available, and so gains could only be obtained by modifying the post-processing procedure rather than training a new model. In the segmentation architecture, the nuclei and cell maps are first obtained via the corresponding predictor U-Net^48^. The next step is the post-processing of the outputs. To improve the efficiency of HCS, we introduce down-scaling and up-scaling blocks at the start and the end of the process respectively. Since post-processing is largely based on various morphological operations, its complexity is proportional to the product of the processed image dimensions. Therefore, reducing the spatial dimensions by 50% resulted in a speedup of a factor of at least 2. Another effect of the reduced dimensionality was a large reduction in high-frequency noise. This allowed simplification of the pipeline by removing extra processing in two fine-tuning blocks and in the Segmentation and gradual object removal block. These changes resulted in a 2x speed-up. However, such speed improvement resulted in a deterioration in system accuracy of around 0.2% mAP.

### DSA, CLH, and CLA training and inference configurations

The DSA comprises a baseline DNN followed by the dual stream network. From each image, *N* cells are selected, resized and flattened as a batch (typically *N* = 20). Let ${{{{{{{\bf{X}}}}}}}}\in {{\mathbb{R}}}^{A\times B\times 4}$ denote a cell image of resolution *A* × *B*. Each cell **X**, is processed by a base DNN, which embeds an input into the space of compact deep features. The output tensor of the final convolutional layer, denoted as ${{{{{{{\bf{R}}}}}}}}\in {{\mathbb{R}}}^{W\times H\times D}$, is forwarded to a learnable Weibull activation layer^19^, where *W* and *H* are the width and height of the feature map and *D* is the feature dimensionality. The output of the activation layer is forwarded to a Global Average-Pooling (GAP) layer and power-normalisation layer to generate global descriptors, which are then passed to the image stream and cell stream.

The image stream applies Global Max-Pooling to a bag of *N* cell descriptors originating from a single image to generate a unified image representation *V*, which is then passed to a fully connected layer *FC*_1_ and Softmax to generate an image-level prediction. The cell stream takes *N* cell descriptors as an input and outputs the predictions for each cell using a fully-connected layer *FC*_2_ and Softmax. The predictions from the image stream are passed to classification loss layer. The loss layer computes the weighted Binary Cross-Entropy loss (*L*_1_) between the image label and bag prediction. Similarly, the cell stream’s weighted Binary Cross-Entropy loss *L*_2_ is calculated between cell predictions and cell labels. The final loss (*L*_*f*_) is the weighted sum of cell stream loss and image stream loss *L*_*f*_ = *W*_1_ × *L*_1_ + *W*_2_ × *L*_2_. In the first round of DSA training, the cell-level labels are weak, and we therefore intuitively assign a much lower weight to cell stream loss compared with image stream loss. The final loss is computed as *L*_*f*_ = 1 × *L*_1_ + 0.2 × *L*_2_ In the second round, the DSA is trained on improved cell labels obtained from our CRA algorithm. Since we now have more trust in the cell-level labels the final loss is computed as *L*_*f*_ = 1 × *L*_1_ + 1 × *L*_2_. The DSA is trained using an Adam optimiser and cosine annealing learning rate scheduler. The Cell-level Hybrid takes cell images as an input and outputs convolutional features denoted as ${{{{{{{\bf{R}}}}}}}}\in {{\mathbb{R}}}^{W\times H\times D}$. The features are aggregated using Global Average Pooling (GAP) layer and forwarded to classification module (fully-connected layer and Softmax).

In Cell-level Actnet, the convolutions features extracted from cell images are passed to learnable Weibull activation pooling. The transformed features are aggregated using GAP and power-normalisation layers and forwarded to the classification module.

The training of CLH and CLA is performed using weighted Binary Cross-Entropy loss, Focal loss, Adam optimiser, and a cosine annealing scheduler with initial learning rate 2*e*^−4^.

We applied data augmentation in the form of random cropping, flipping, shifting, rotation, scaling and cutout to train all models.

### The Weibull activation layer

The output tensor of the final convolutional layer of DSA or CLA, denoted as ${{{{{{{\bf{R}}}}}}}}\in {{\mathbb{R}}}^{W\times H\times D}$, is forwarded to a learnable activation layer^19^, where *W* and *H* are the width and height of the feature map and *D* is the feature dimensionality. The Weibull activation layer is aimed at maximising the Signal-to-Noise ratio (SNR) of the last convolutional feature map by applying the Weibull function to the tensor **R**, the output tensor of the final convolutional layer. Each element of the tensor **R** is transformed by the Weibull function resulting in the output tensor ${{{{{{{\bf{T}}}}}}}}\in {{\mathbb{R}}}^{W\times H\times D}$ (where 0≤*i* < *W* × *H* × *D*):

2 $${{{{{{{{\bf{T}}}}}}}}}_{i}={\left(\frac{{{{{{{{{\bf{R}}}}}}}}}_{i}}{\lambda }\right)}^{\zeta -1}\exp (-{({{{{{{{{\bf{R}}}}}}}}}_{i}/\gamma )}^{\eta }).$$

The learnable parameters of the activation layer are *λ*, *ζ*, *γ*, and *η*. The output of the activation layer is fed to the Global Average Pooling (GAP) layer, denoted as *P*(**T**), to compute the global vector **S**:

3 $${{{{{{\mathbf{S}}}}}}} = \left( \frac{1}{WH} \mathop{\sum}\limits^{W}_{i}\mathop{\sum}\limits^{H}_{j} T_{ijk} \right)_{k = 1}^D$$

Each element (*s* ∈ **S**) of the tensor **S** is power normalised to balance the non-linear scaling of the Weibull function. The power-normalisation function is represented as $\delta :{{\mathbb{R}}}^{D}\to {{\mathbb{R}}}^{D}$, with the rule:

4 $$\delta (s)=\alpha {s}_{1}^{\beta },\alpha {s}_{2}^{\beta },...,\alpha {s}_{D}^{\beta }$$

where *α*, *β* are learnable scaling parameters.

### Experimental evaluation

We followed the bestfitting solution code (https://github.com/topics/hpa-challenge-2021) and the algorithm description^11,33^ to retrain the models without using the antibody-id information. The base model for the bestfitting solution, Fair Cell Activation Network (FCAN), was trained to jointly minimise three loss functions: reconstructing regularisation loss between the image and cells Class Activation Maps (CAMs), classification losses supervised by nineteen class labels and metric learning losses supervised by antibody-ids. We removed the metric learning loss layer and retrained the FCAN using the original hyperparameters. Following this, we used the original post-processing algorithm and an ensemble of FCANs and cell-level models to obtain the final solution.

The class-wise performances of Kaggle teams were obtained from Supplementary Tables 1 to 11 of ref. ^11^.

### Statistics and reproducibility

The study did not employ statistical analysis when evaluating the HCPL performance on Kaggle platform. As this is a Kaggle code competition, all code submission for inference were collected and graded automatically, which allows for reproduction of the scores. All training data and HPAv20 dataset are publicly available for model training and testing dataset is available on Kaggle platform. Results reported for the HCPL system can be reproduced using the standard submission process for the Human Protein Atlas - Single Cell Classification Kaggle competition^12^ and by utilising the source code and models provided in the Code Availability section. All the data required to reproduce the figures are in Figshare 10.6084/m9.figshare.22251208.

### Reporting summary

Further information on research design is available in the Nature Portfolio Reporting Summary linked to this article.

## Supplementary information

Husain_Peer Review File

Supplementary Information

Reporting Summary

latex_source_files

## Data Availability

The HPA training dataset analysed during the current study are available from Kaggle, https://www.kaggle.com/competitions/hpa-single-cell-image-classification/data. The Bad Cell dataset generated during the current study are available at https://www.kaggle.com/datasets/anokas/hpabadcellxgboost. The HPA test set is not openly available and was not accessible to the authors; code submissions can be made for scoring through the Kaggle page.

## Code availability

The Python source code for HCPL and model weights suitable for inference are available online via the project’s web page at https://bit.ly/hpa-hcpl.
