# Supplementary material for: Single-cell subcellular protein localisation using novel ensembles of diverse deep architectures
Source: Commun Biol. 2023 May 5;6:489. doi: 10.1038/s42003-023-04840-z (PMC10163260; doi:10.1038/s42003-023-04840-z)
Supplement: Supplementary file 2 — Supplementary Information [file 42003_2023_4840_MOESM2_ESM.pdf]

Supplementary information

Single-cell Subcellular Protein Localisation Using Novel Ensembles of Diverse Deep Architectures

Syed Sameed Husain[1*], Eng-Jon Ong[1], Dmitry Minskiy[1], Mikel Bober-Irizar[1,2], Amaia Irizar[2] and Miroslaw Bober[1,2]

[1]CVSSP, University of Surrey, Guildford, GU27XH, Surrey, UK.
[2]ForecomAI, Leatherhead, KT228QY, Surrey, UK.

*Corresponding author(s). E-mail(s): sameed.husain@surrey.ac.uk;
Contributing authors: e.ong@surrey.ac.uk; d.minskiy@surrey.ac.uk; mikel@mxbi.net; a.irizar@forecom.ai; m.bober@surrey.ac.uk;

Supplementary Table 1. Human Protein Atlas - Single Cell Classification dataset Labels-ids, Label names and short Label Names

Class	LabelName	ShortLabelName
0	Nucleoplasm	Nucleoplasm
1	Nuclear membrane	Nuc.Membrane
2	Nucleoli	Nucleoli
3	Nucleoli fibrillar	Nuc.Fib.C
4	Nuclear speckles	Nuc.Speckles
5	Nuclear bodies	Nuc.Bodies
6	Endoplasmic reticulum	ER
7	Golgi apparatus	Golgi
8	Intermediate filaments	Int.Fil
9	Actin filaments	Actin.Fil
10	Microtubules	Microtubules
11	Mitotic spindle	M.Spindle
12	Centrosome	Centrosome
13	Plasma membrane	Pl.Membrane
14	Mitochondria	Mitochondria
15	Aggresome	Aggresome
16	Cytosol	Cytosol
17	Vesicles	Ves.Punctate
18	Negative	Negative

Supplementary Note 1. Description of the Human Protein Atlas - Single Cell Classification competition models

The approaches used in the Kaggle competition [1] for classifying individual cells employed deep learning with two distinct models. The first is a Cell-level model (CLM), which takes segmented cells as input and outputs the predictions. An example of CLM is the Bag-of-Cells model [2], where the deep features from N cells in an image (bag) are extracted by a base CNN and aggregated using Mean pooling. The pooled tensor is forwarded to the learnable Attention pooling layer responsible for learning the weights for each cell representation belonging to a particular image: higher weighting is given to discriminative cells compared to the non-discriminative cells. The weighted representations are then pooled using the sum pooling function. Finally, the BCE loss is computed between the image label and image-level prediction. A more data-centric approach to developing CLM [3] involves re-labelling the cells using manual labelling and Meta Pseudo labelling algorithms. The CNN is then trained on refined data using Focal loss.

The disadvantage of CLM is inferior classification performance due to training using weak cell labels. To address this, Multi-head models (MHMs) were developed. The MHMs comprise an image head that models the entire population of cells and a cell head that classifies patterns in each cell in the image. An example of MHM is the Fair Cell Activation Network (FCAN) [4], an improved version of Puzzle-CAM [31], trained to jointly minimise multiple loss functions: reconstructing regularisation loss between the image and cells Class Activation Maps (CAMs), classification

losses supervised by nineteen class labels and metric learning losses supervised by antibody labels. The Duo-branch CNN [5] is another example of MHM, where a random sample of N cells is first extracted from an image and then forwarded to a CNN. The last convolutional features from the CNN are aggregated and fed to an image head and a cell head. The fully connected (FC) layer of the cell head generates probabilities for each cell, while the FC layer of the image head outputs a single image probability. Finally, the predictions from two heads are passed to respective Binary Cross Entropy losses, and the final loss is the sum of losses from two heads. Another approach is the modified Puzzle-CAM [6] which consists of a Siamese network with reconstructing regularization loss that reduces the differences between the CAM of the original image and merged CAMs of image patches.

References

[1] Le, T. *et al.* Analysis of the human protein atlas weakly supervised single-cell classification competition. *Nat. methods* **19**, 1221–1229 (2022).

[2] Henkel, C. *Third place Dieter part for Human Protein Atlas - Single Cell Classification*. https://www.kaggle.com/competitions/hpa-single-cell-image-classification/discussion/238898 (2021).

[3] Banić, N. *Fourth Place Solution for Human Protein Atlas - Single Cell Classification*. https://www.kaggle.com/competitions/hpa-single-cell-image-classification/discussion/239071 (2021).

[4] Dai, S. *Fair Cell Activation Network and Swin Transformer, the first place solution for Human Protein Atlas - Single Cell Classification*. https://www.kaggle.com/competitions/hpa-single-cell-image-classification/ (2021).

[5] Makarov, I. *Second Place Solution for Human Protein Atlas - Single Cell Classification*. https://www.kaggle.com/competitions/hpa-single-cell-image-classification/discussion/238645 (2021).

[6] Benhamou, E. *Third place solution MPWARE part for Human Protein Atlas - Single Cell Classification*. https://www.kaggle.com/competitions/hpa-single-cell-image-classification/discussion/238862 (2021).
