# Supplementary material for: Single-cell subcellular protein localisation using novel ensembles of diverse deep architectures
Source: Commun Biol. 2023 May 5;6:489. doi: 10.1038/s42003-023-04840-z (PMC10163260; doi:10.1038/s42003-023-04840-z)
Supplement: Supplementary file 3 — Reporting Summary [file 42003_2023_4840_MOESM3_ESM.pdf]

nature portfolio

Corresponding author(s): Syed Sameed Husain
Last updated by author(s): Apr 10, 2023

Reporting Summary

Nature Portfolio wishes to improve the reproducibility of the work that we publish. This form provides structure for consistency and transparency in reporting. For further information on Nature Portfolio policies, see our Editorial Policies and the Editorial Policy Checklist.

Statistics

For all statistical analyses, confirm that the following items are present in the figure legend, table legend, main text, or Methods section.

n/a	Confirmed	
☐	☒	The exact sample size (*n*) for each experimental group/condition, given as a discrete number and unit of measurement
☐	☒	A statement on whether measurements were taken from distinct samples or whether the same sample was measured repeatedly
☒	☐	The statistical test(s) used AND whether they are one- or two-sided<br>*Only common tests should be described solely by name; describe more complex techniques in the Methods section.*
☐	☒	A description of all covariates tested
☐	☒	A description of any assumptions or corrections, such as tests of normality and adjustment for multiple comparisons
☒	☐	A full description of the statistical parameters including central tendency (e.g. means) or other basic estimates (e.g. regression coefficient) AND variation (e.g. standard deviation) or associated estimates of uncertainty (e.g. confidence intervals)
☒	☐	For null hypothesis testing, the test statistic (e.g. *F*, *t*, *r*) with confidence intervals, effect sizes, degrees of freedom and *P* value noted<br>*Give P values as exact values whenever suitable.*
☒	☐	For Bayesian analysis, information on the choice of priors and Markov chain Monte Carlo settings
☒	☐	For hierarchical and complex designs, identification of the appropriate level for tests and full reporting of outcomes
☒	☐	Estimates of effect sizes (e.g. Cohen's *d*, Pearson's *r*), indicating how they were calculated

Our web collection on statistics for biologists contains articles on many of the points above.

Software and code

Policy information about availability of computer code

Data collection	Our work uses the HPA dataset provided by the ``Human Protein Atlas - Single Cell Classification'' Kaggle challenge. This consists of images from the freely accessible Human Protein Atlas project. The dataset is available at: https://www.kaggle.com/c/hpa-single-cell-image-classification. The collection of data is described in detail in https://doi.org/10.1038/s41592-022-01606-z. The dataset for our Visual Integrity Detector System is available at https://www.kaggle.com/datasets/anokas/hpabadcellxgboost
Data analysis	For the training of our models we mainly used Python 3 (with libraries such as numpy 1.19.5, pandas 1.1.5, scikit-image 0.17.2, scikit-learn 0.24.1, scipy 1.5.4, matplotlib 3.3.4, opencv 4.5.3.56, torch 1.7.1). The evaluation of models is performed on Kaggle private leaderboard: https://www.kaggle.com/competitions/hpa-single-cell-image-classification/submissions. The Python code for HCPL and model weights suitable for inference are available online via the project's web page at https://bit.ly/hpa-hcpl

For manuscripts utilizing custom algorithms or software that are central to the research but not yet described in published literature, software must be made available to editors and reviewers. We strongly encourage code deposition in a community repository (e.g. GitHub). See the Nature Portfolio guidelines for submitting code & software for further information.

Data

Policy information about availability of data

All manuscripts must include a data availability statement. This statement should provide the following information, where applicable:

- Accession codes, unique identifiers, or web links for publicly available datasets
- A description of any restrictions on data availability
- For clinical datasets or third party data, please ensure that the statement adheres to our policy

The primary dataset used for this study is freely available at: https://www.kaggle.com/c/hpa-single-cell-image-classification/data.
The additional dataset HPAv20 is publicly available on the Human Protein Atlas: https://v20.proteinatlas.org/

Human research participants

Policy information about studies involving human research participants and Sex and Gender in Research.

Reporting on sex and gender	*Use the terms sex (biological attribute) and gender (shaped by social and cultural circumstances) carefully in order to avoid confusing both terms. Indicate if findings apply to only one sex or gender; describe whether sex and gender were considered in study design whether sex and/or gender was determined based on self-reporting or assigned and methods used. Provide in the source data disaggregated sex and gender data where this information has been collected, and consent has been obtained for sharing of individual-level data; provide overall numbers in this Reporting Summary. Please state if this information has not been collected. Report sex- and gender-based analyses where performed, justify reasons for lack of sex- and gender-based analysis.*
Population characteristics	*Describe the covariate-relevant population characteristics of the human research participants (e.g. age, genotypic information, past and current diagnosis and treatment categories). If you filled out the behavioural & social sciences study design questions and have nothing to add here, write "See above."*
Recruitment	*Describe how participants were recruited. Outline any potential self-selection bias or other biases that may be present and how these are likely to impact results.*
Ethics oversight	*Identify the organization(s) that approved the study protocol.*

Note that full information on the approval of the study protocol must also be provided in the manuscript.

Field-specific reporting

Please select the one below that is the best fit for your research. If you are not sure, read the appropriate sections before making your selection.

☒ Life sciences ☐ Behavioural & social sciences ☐ Ecological, evolutionary & environmental sciences

For a reference copy of the document with all sections, see nature.com/documents/nr-reporting-summary-flat.pdf

Life sciences study design

All studies must disclose on these points even when the disclosure is negative.

Sample size	Sample size of training and testing dataset was detailed in Methods. Our work uses the HPA dataset provided by the ``Human Protein Atlas - Single Cell Classification'' Kaggle challenge. This consists of images from the freely accessible Human Protein Atlas project. A total of 104307 images (internal and external dataset) were made available for training purposes whilst an additional 1776 unseen images were retained by the challenge organisers for testing purposes, further split into 559 images for the public leaderboard and 1217 images for the private leaderboard.
Data exclusions	For training and testing datasets, inclusions and exclusion criteria were described in Manuscript. Our models are trained using only the training dataset without visibility of the test set and without external datasets. We used scores on the public test set to develop models and report performance on the private test set.
Replication	As this is a Kaggle code competition, all code submission for inference were collected and graded automatically, which allows for reproduction of the scores. All training data and HPAv20 dataset are publicly available for model training and testing dataset is available on Kaggle platform for replication of the performance. Results reported for the HCPL system can be reproduced using the standard submission process for the ”Human Protein Atlas - Single Cell Classification” Kaggle competition and by utilizing the source code and models provided in the ”Code Availability” section.
Randomization	Test set randomization criteria is described in Nature Methods article: https://doi.org/10.1038/s41592-022-01606-z. Annotation of cells in the test set was done individually, in random order and assigned randomly to annotators to avoid bias.
Blinding	The authors do not have access to any cell labels in all datasets as well any part of the private test set, ensuring a fair evaluation. For a fair

Blinding	comparison, we only used the public leaderboard to develop our algorithm and avoided any optimisation based on the private leaderboard. Once we finalised the architecture based on the public leaderboard, we reported the performance on the private leaderboard. As per the competition rules, the model scoring was performed by Kaggle without the authors' intervention: https://www.kaggle.com/competitions/hpa-single-cell-image-classification/leaderboard

Reporting for specific materials, systems and methods

We require information from authors about some types of materials, experimental systems and methods used in many studies. Here, indicate whether each material, system or method listed is relevant to your study. If you are not sure if a list item applies to your research, read the appropriate section before selecting a response.

Materials & experimental systems

n/a	Involved in the study
☒	☐ Antibodies
☒	☐ Eukaryotic cell lines
☒	☐ Palaeontology and archaeology
☒	☐ Animals and other organisms
☒	☐ Clinical data
☒	☐ Dual use research of concern

Methods

n/a	Involved in the study
☒	☐ ChIP-seq
☒	☐ Flow cytometry
☒	☐ MRI-based neuroimaging
