# Supplementary material for: Single-cell subcellular protein localisation using novel ensembles of diverse deep architectures
Source: Commun Biol. 2023 May 5;6:489. doi: 10.1038/s42003-023-04840-z (PMC10163260; doi:10.1038/s42003-023-04840-z)
Supplement: Supplementary file 4 — latex_source_files [file 42003_2023_4840_MOESM4_ESM.zip › Figures/Figure9.pdf]

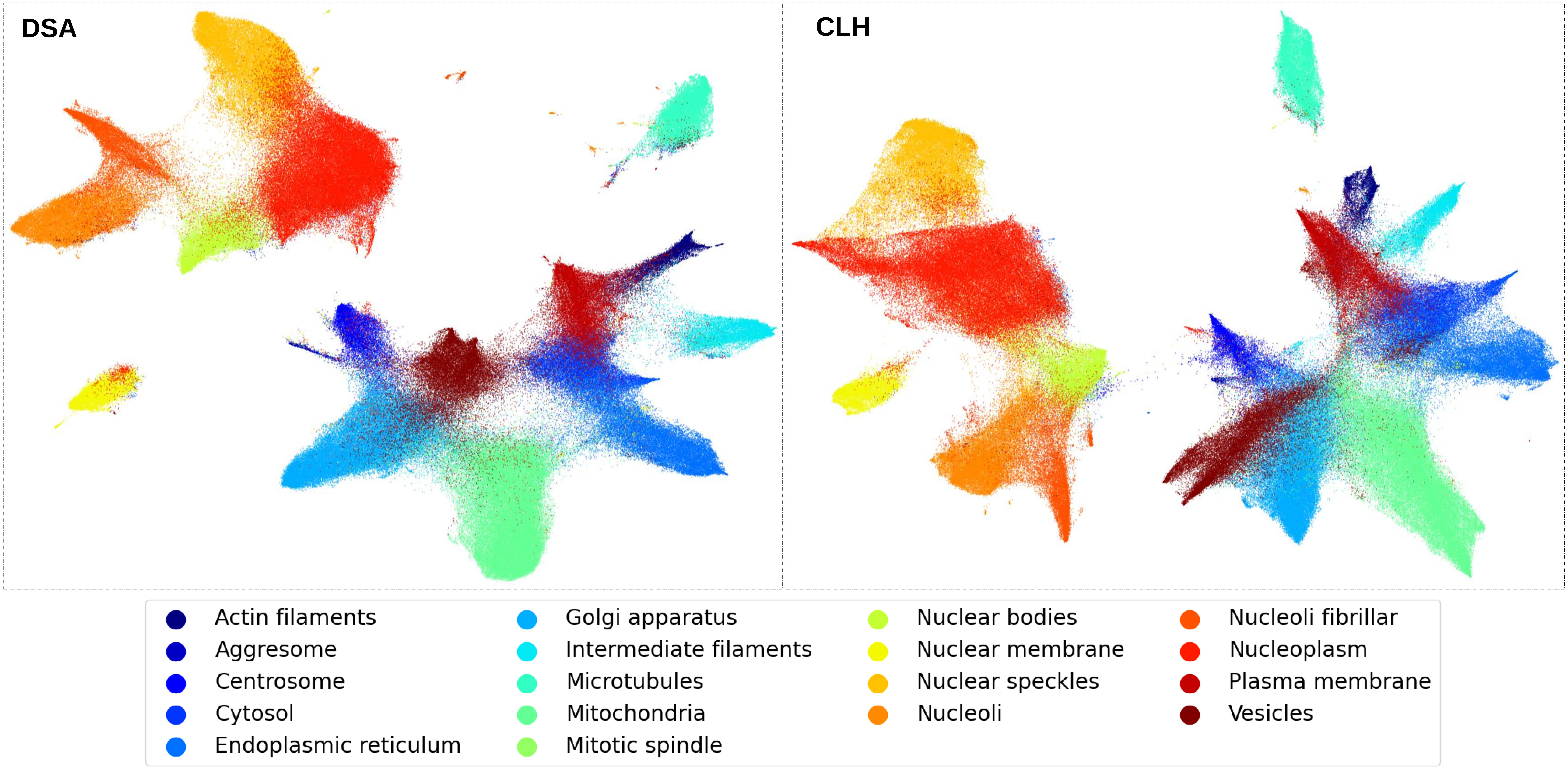
DSA
CLH
Actin filaments
Aggresome
Centrosome
Cytosol
Endoplasmic reticulum
Golgi apparatus
Intermediate filaments
Microtubules
Mitochondria
Mitotic spindle
Nuclear bodies
Nuclear membrane
Nuclear speckles
Nucleoli
Nucleoli fibrillar
Nucleoplasm
Plasma membrane
Vesicles
