# Supplementary figures and images for: Single-cell subcellular protein localisation using novel ensembles of diverse deep architectures
Source: Commun Biol. 2023 May 5;6:489. doi: 10.1038/s42003-023-04840-z (PMC10163260; doi:10.1038/s42003-023-04840-z)

a

## Overview of dataset and the HPA challenge

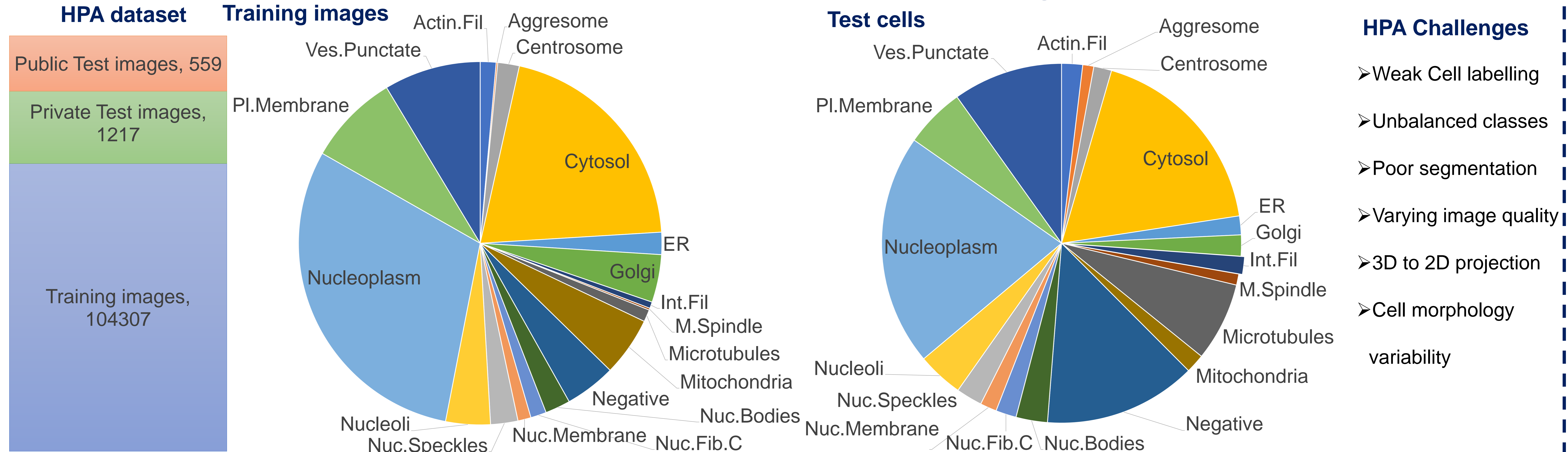

b

## Hybrid subCellular Protein Localiser (HCPL)

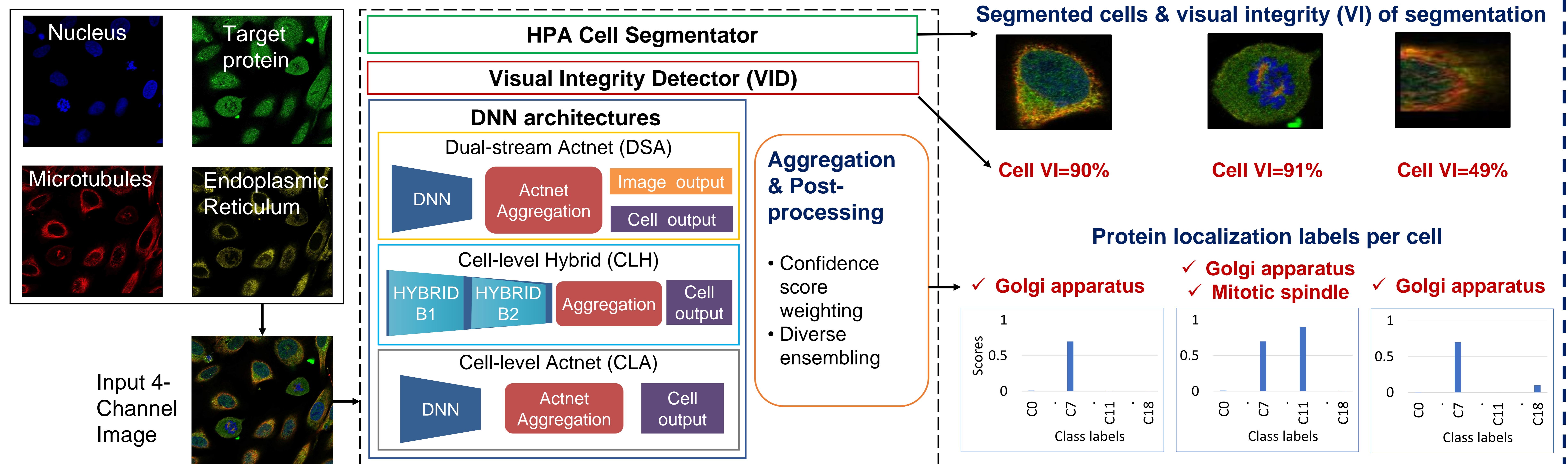

Supplement: Supplementary file 4 — latex_source_files [file 42003_2023_4840_MOESM4_ESM.zip › Figures/Figure1.pdf]

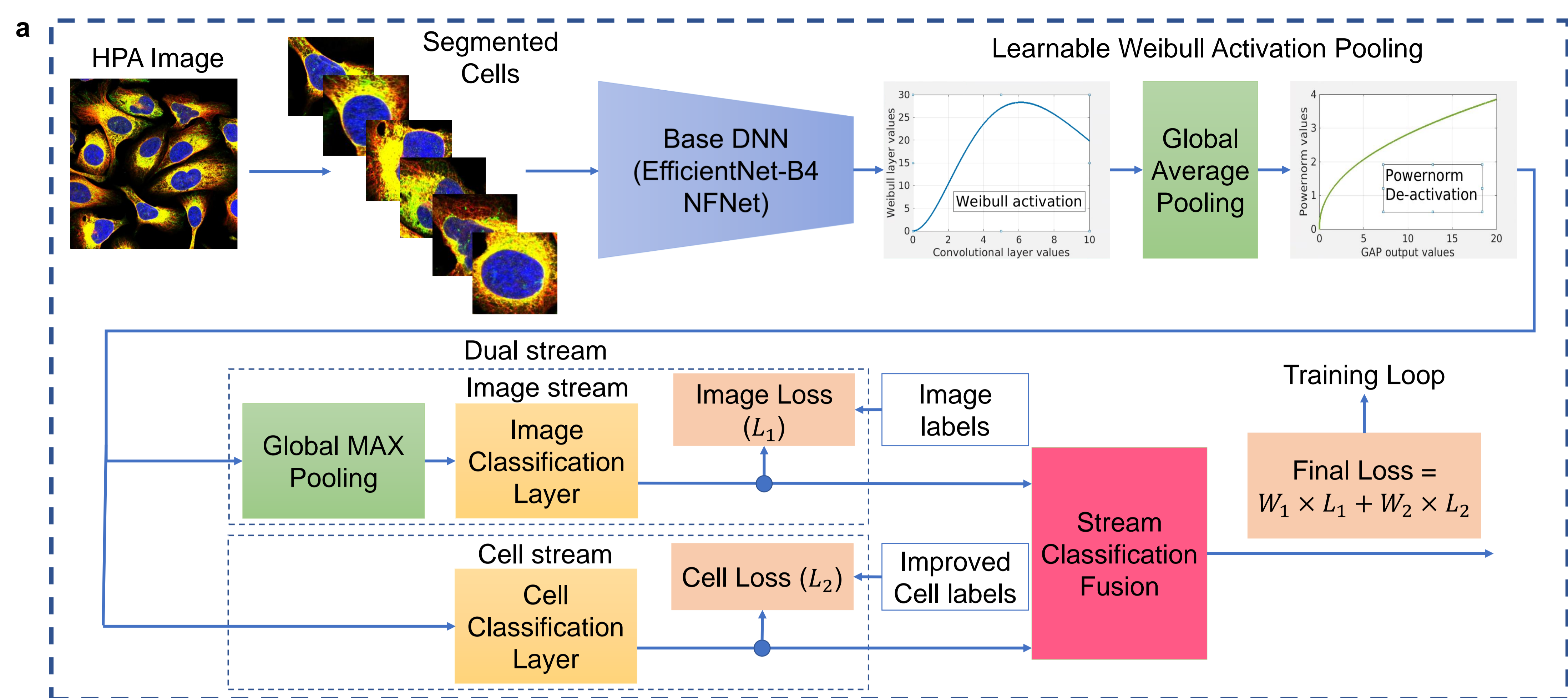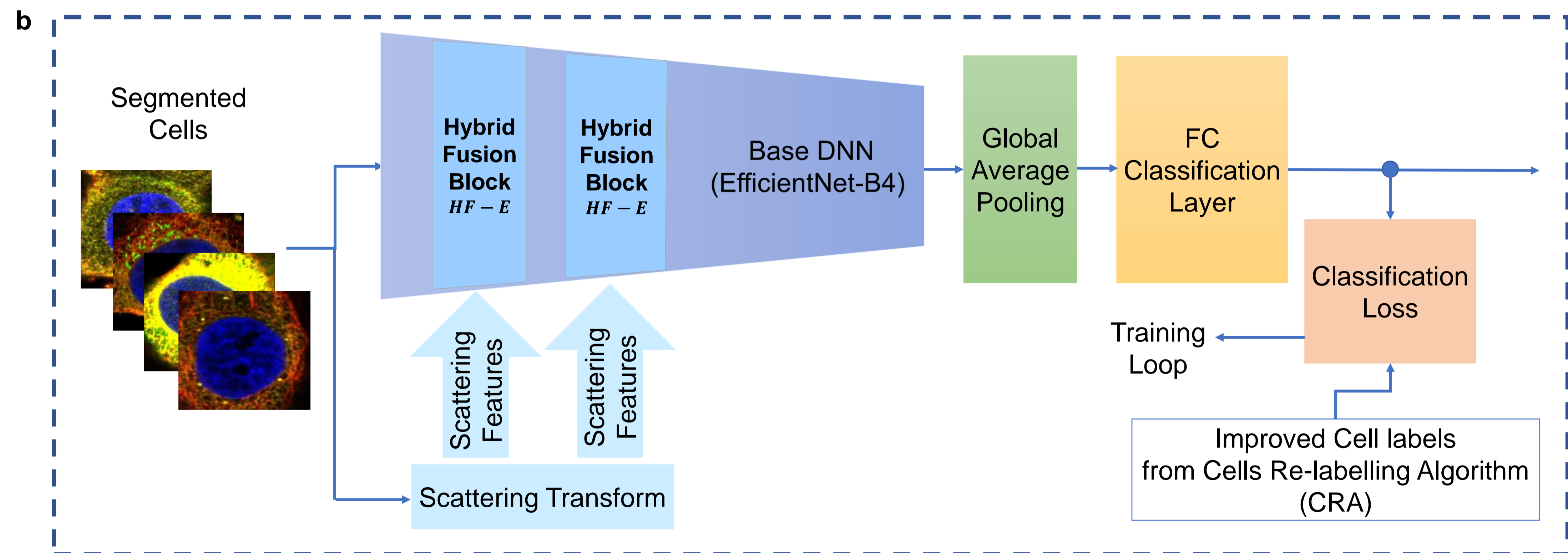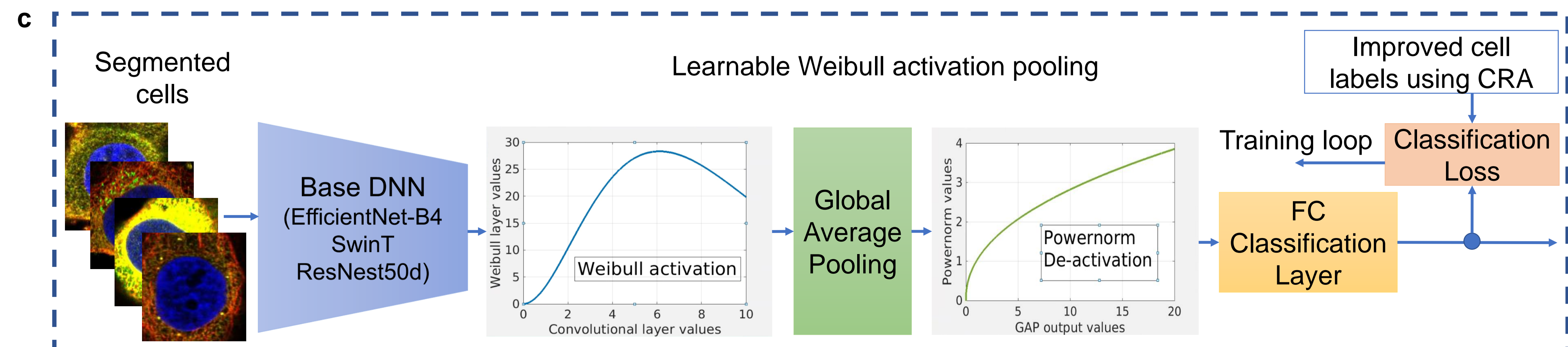

Supplement: Supplementary file 4 — latex_source_files [file 42003_2023_4840_MOESM4_ESM.zip › Figures/Figure2.pdf]

**a**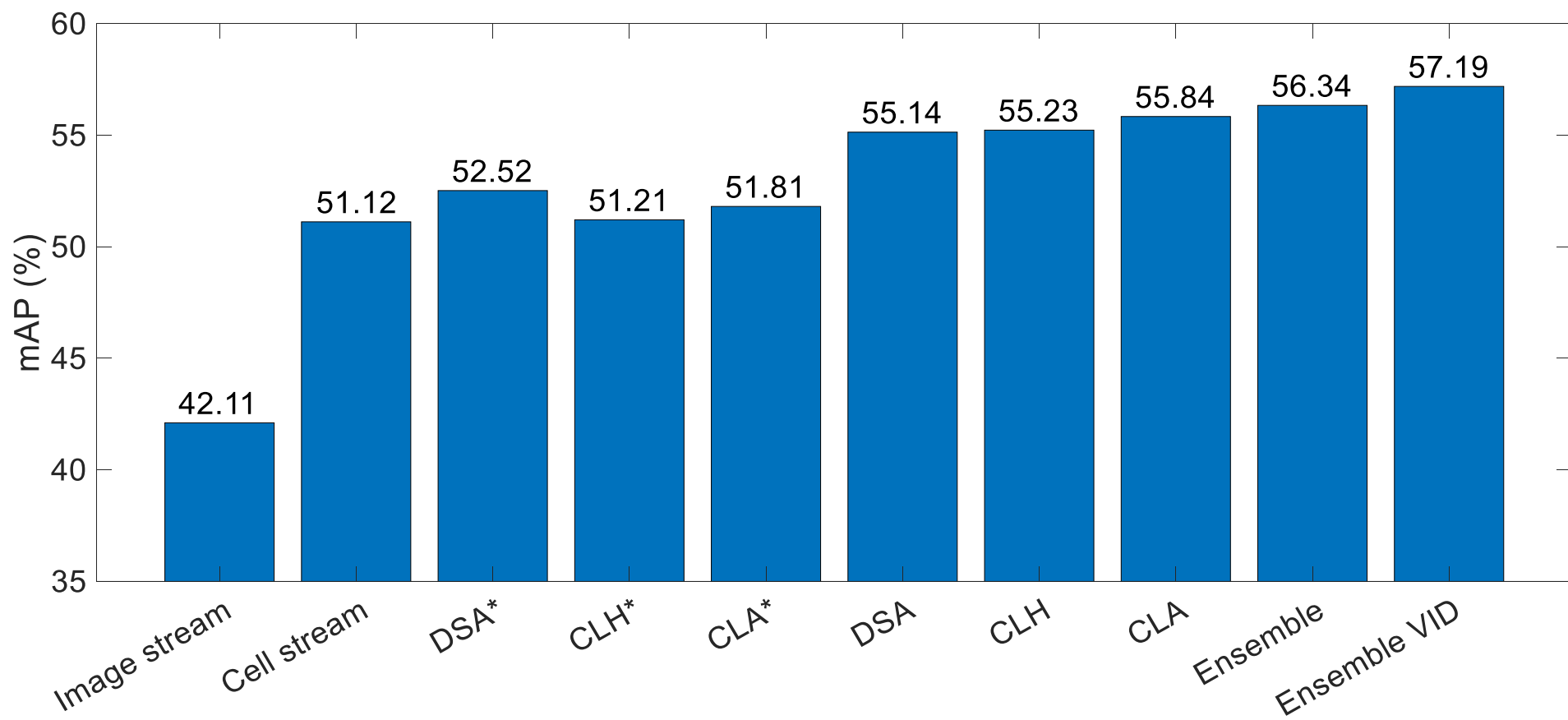**b**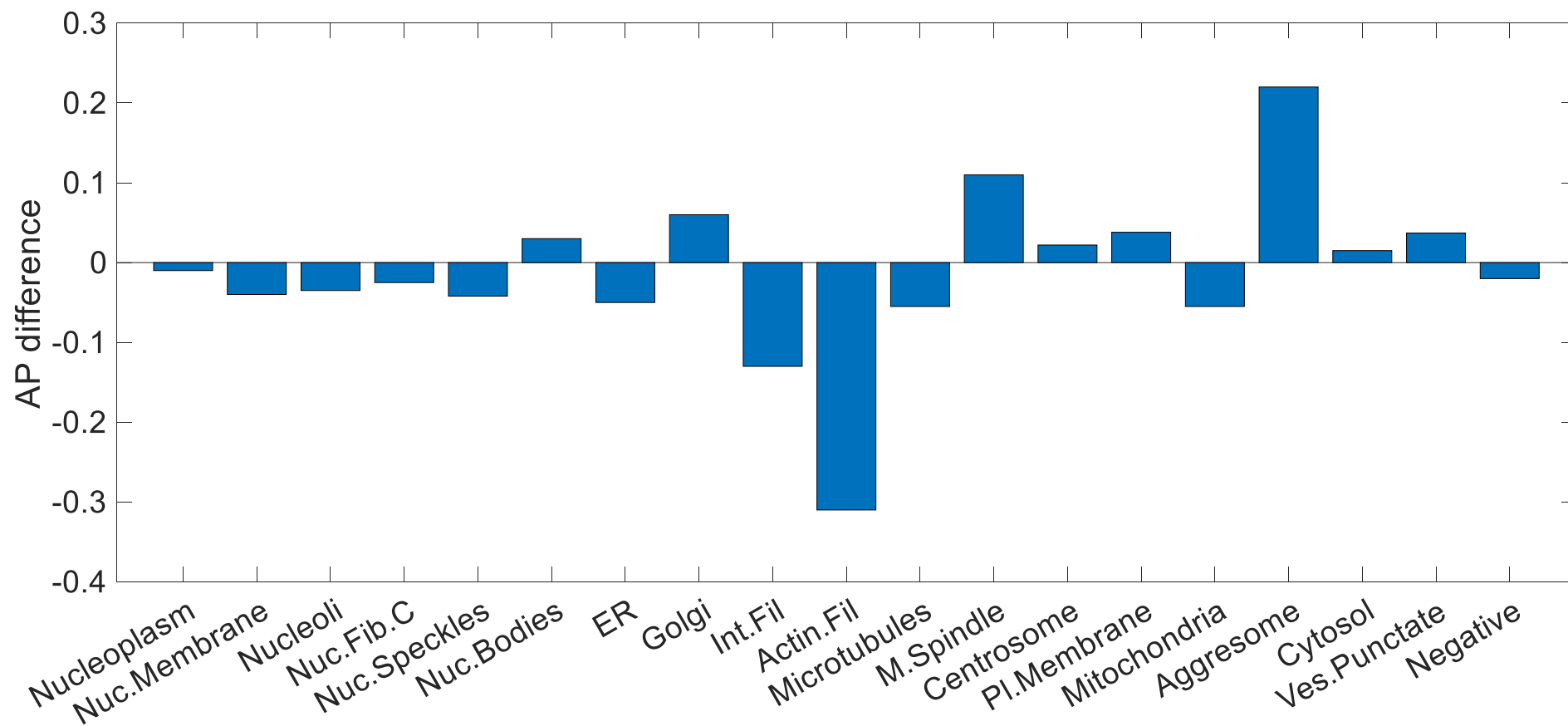

Supplement: Supplementary file 4 — latex_source_files [file 42003_2023_4840_MOESM4_ESM.zip › Figures/Figure3.pdf]

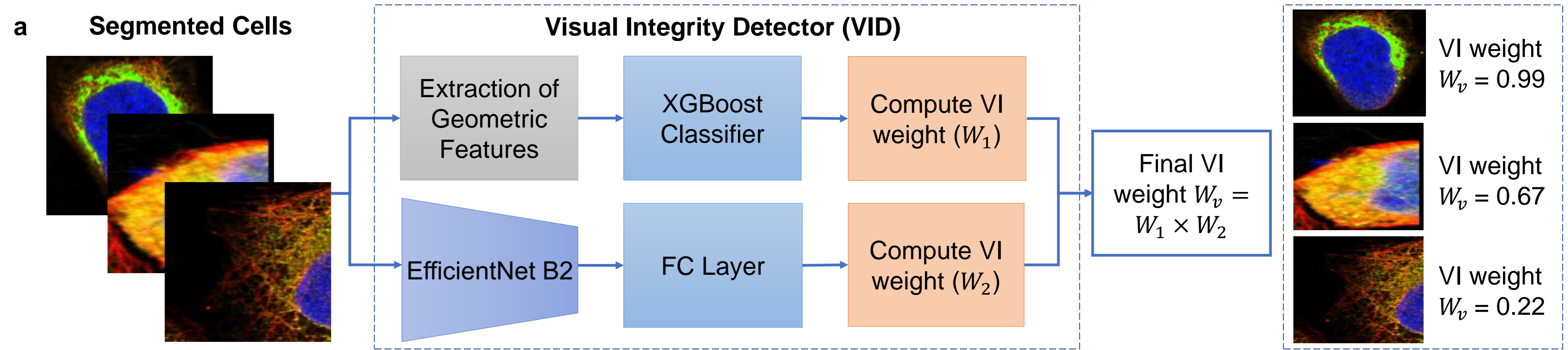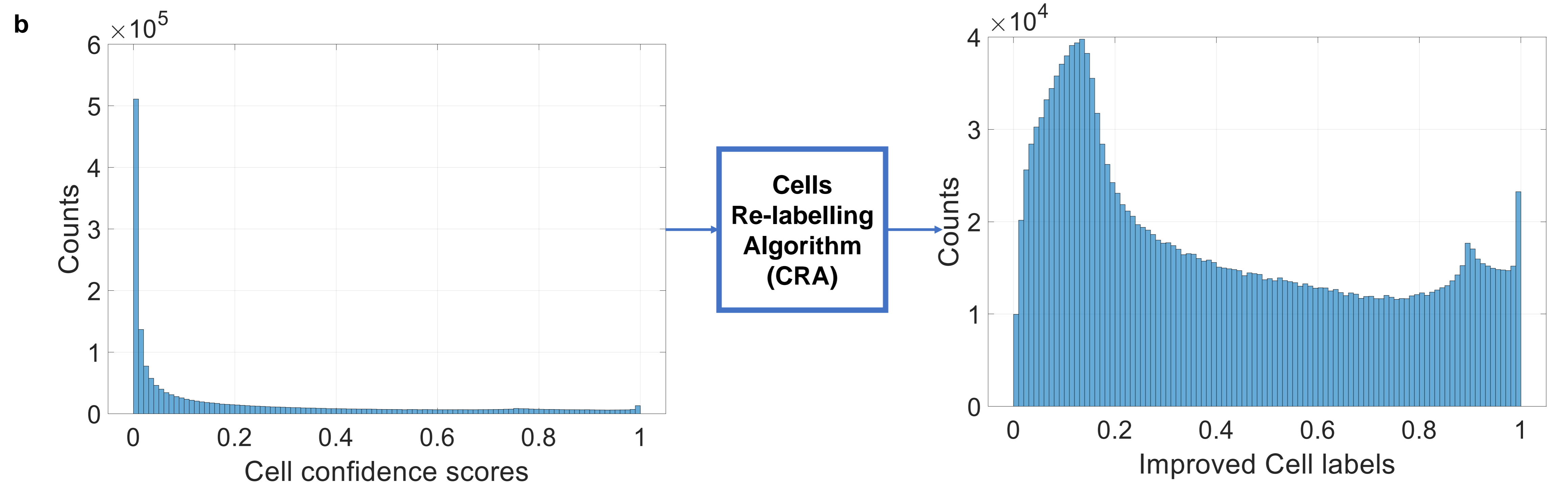

Supplement: Supplementary file 4 — latex_source_files [file 42003_2023_4840_MOESM4_ESM.zip › Figures/Figure4.pdf]

a

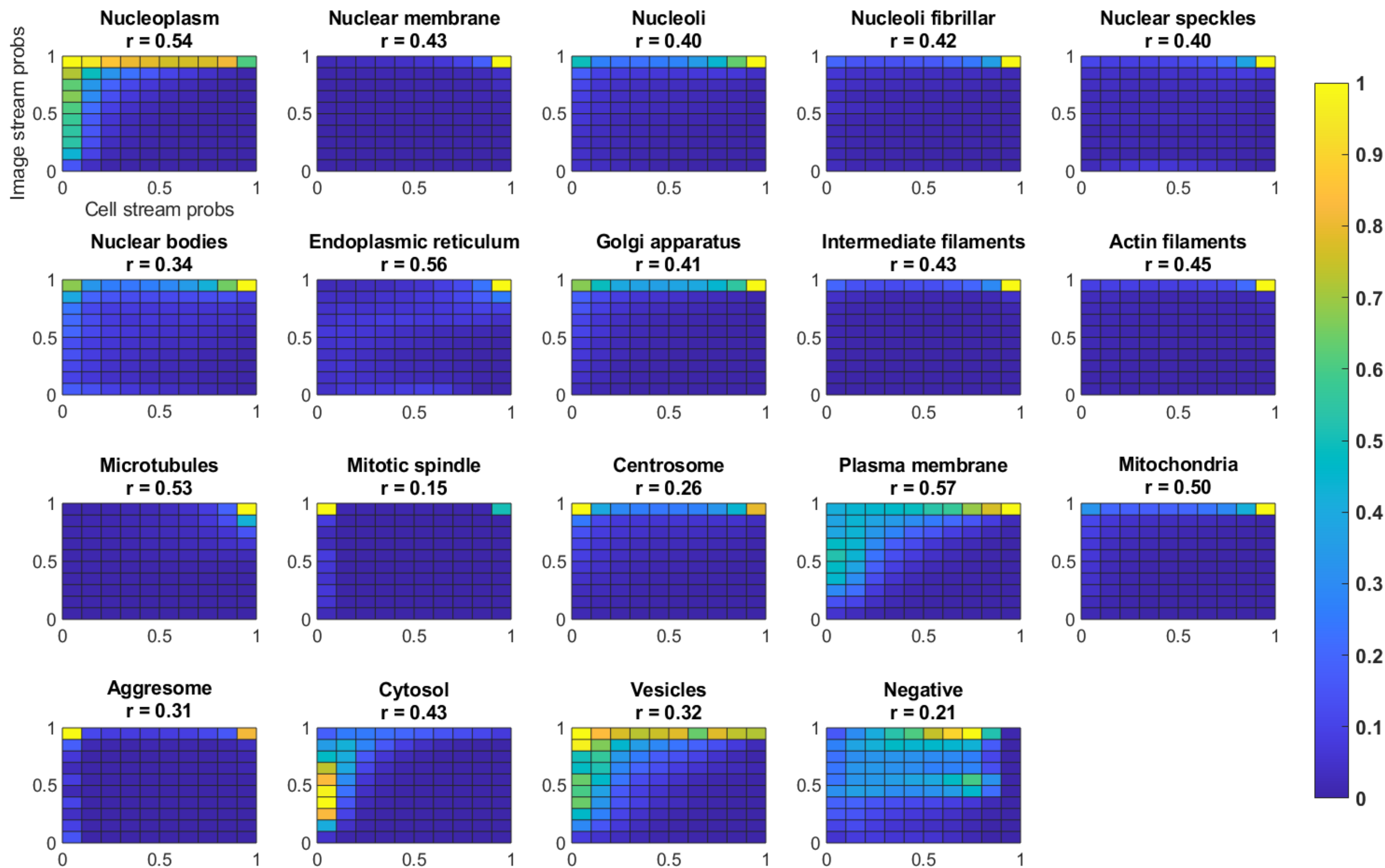

b

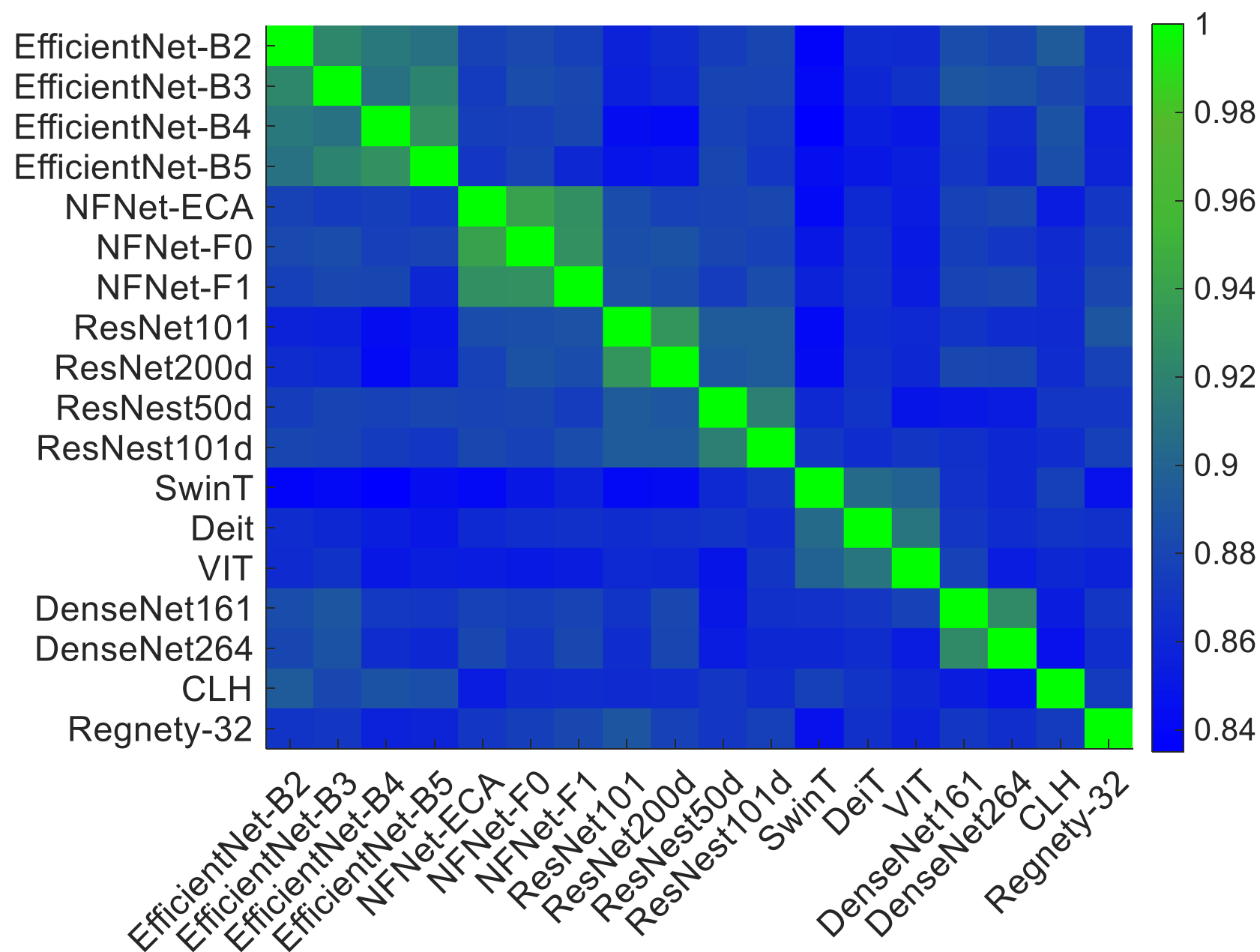

c

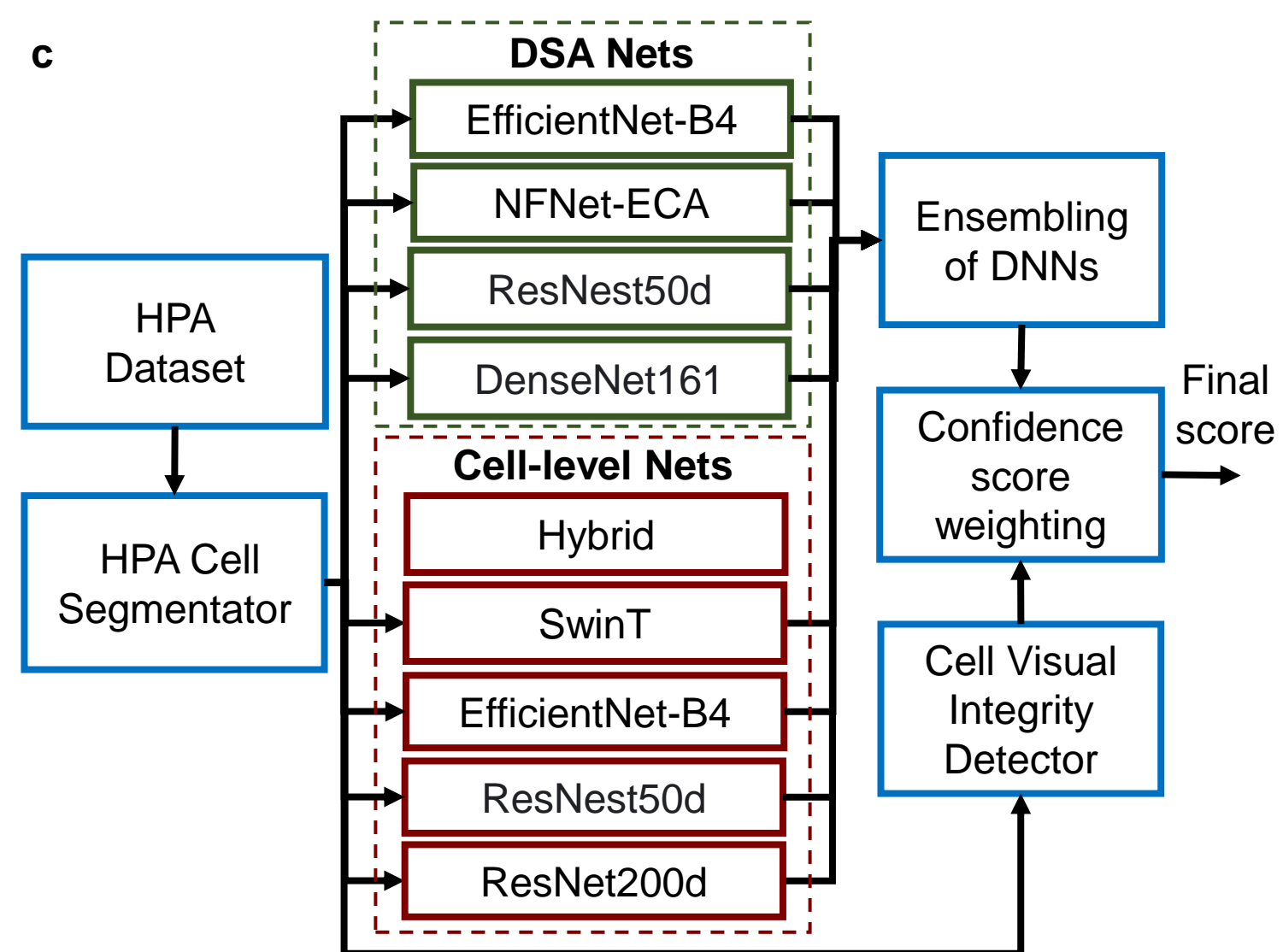

Supplement: Supplementary file 4 — latex_source_files [file 42003_2023_4840_MOESM4_ESM.zip › Figures/Figure5.pdf]

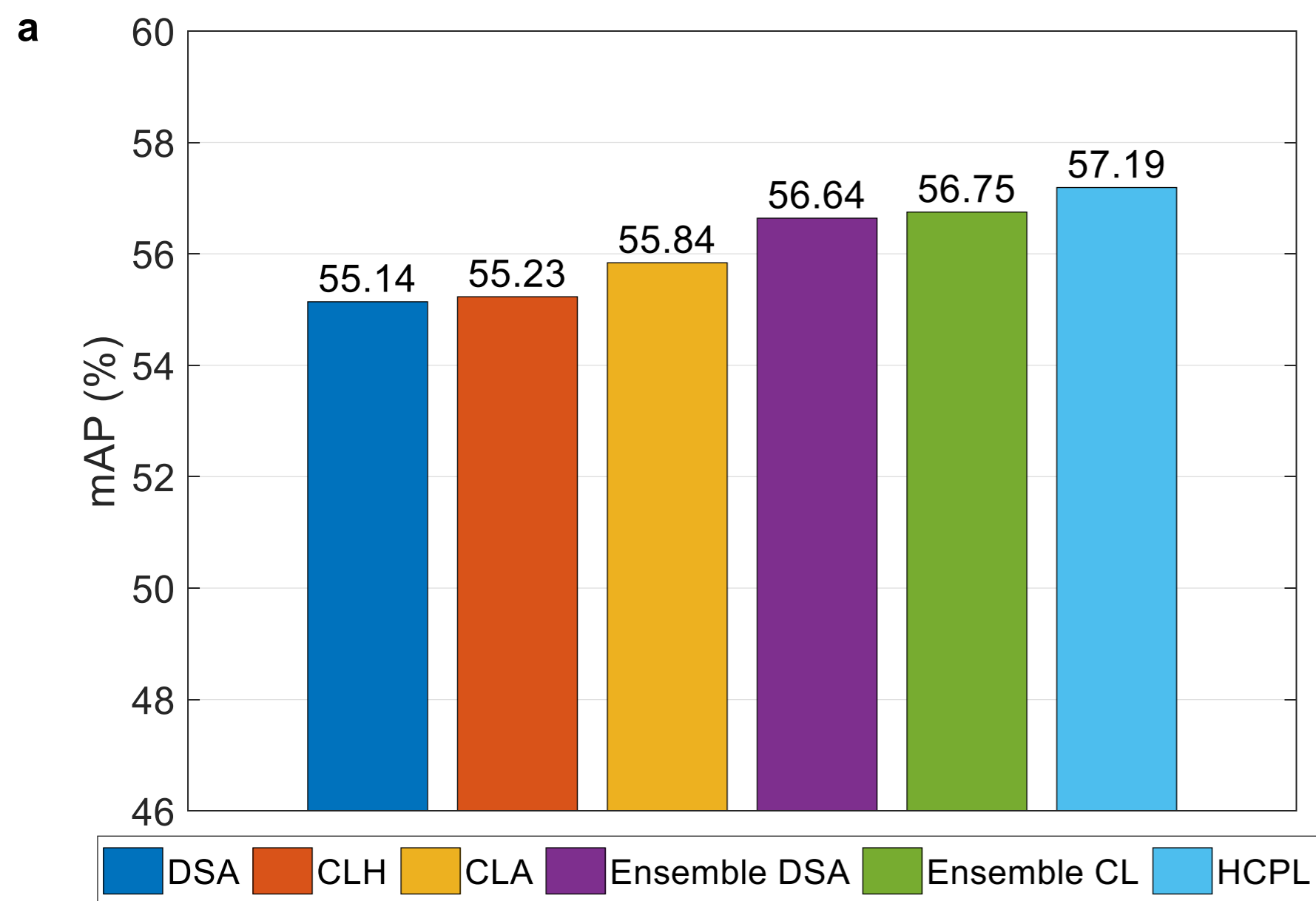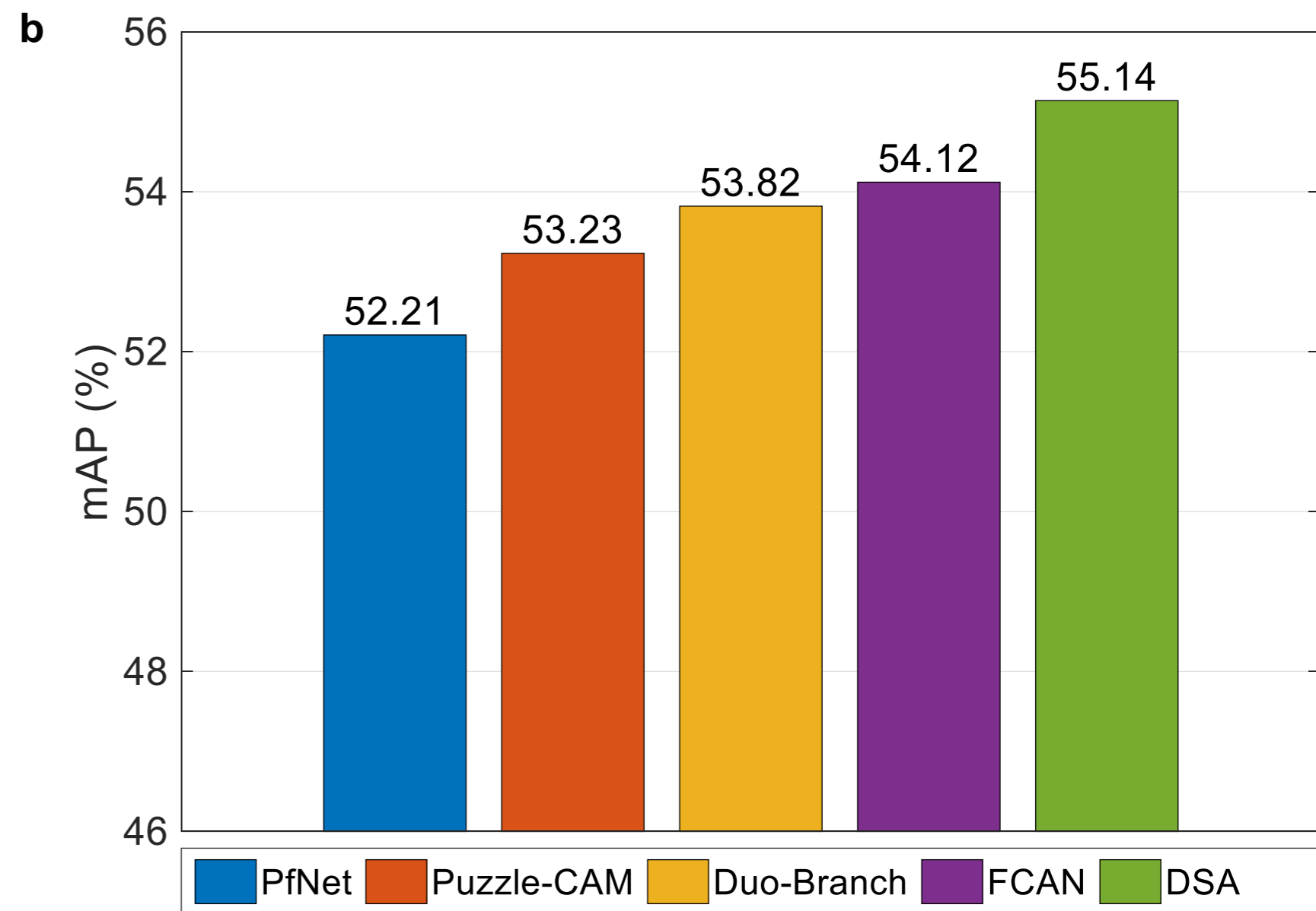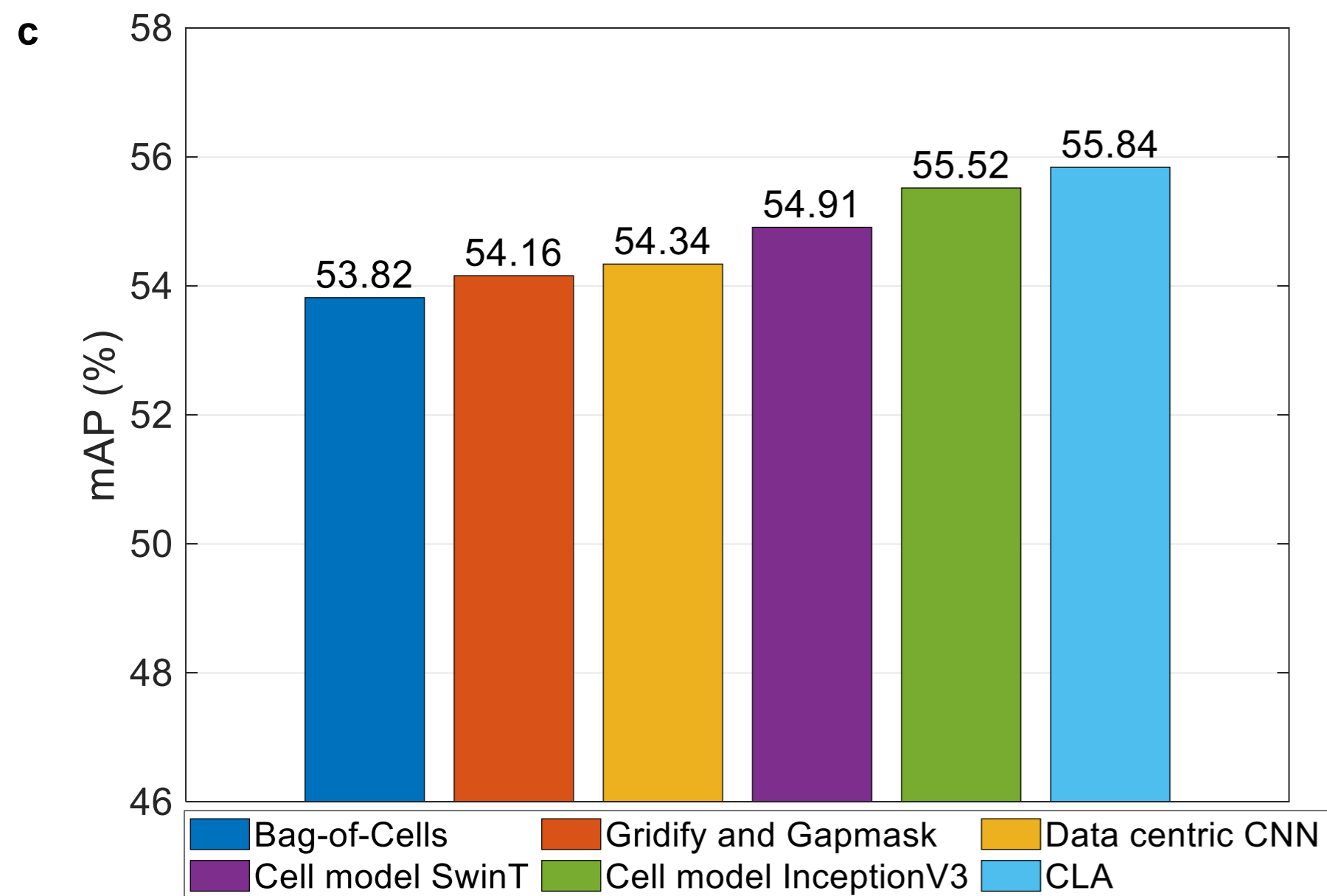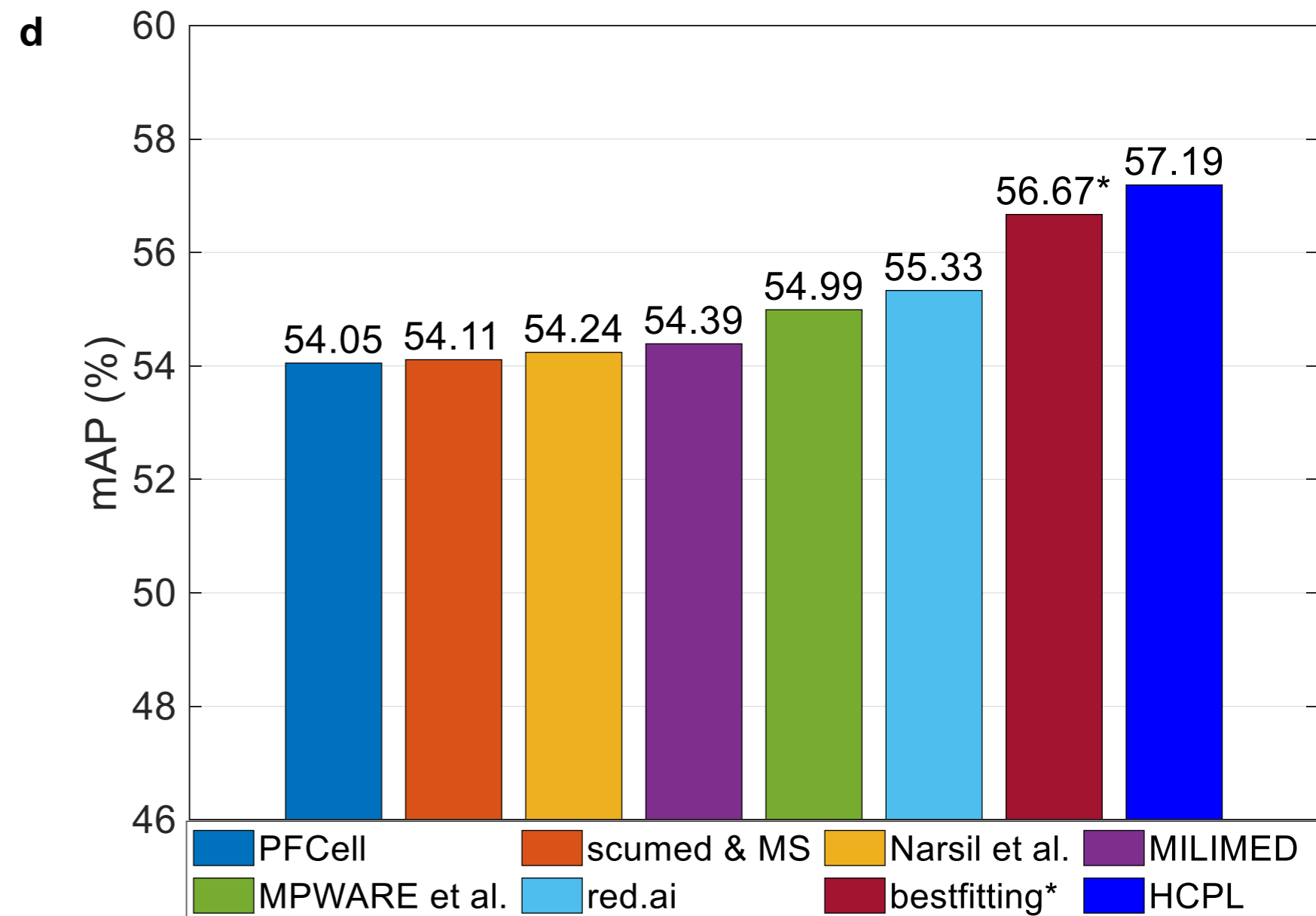

Supplement: Supplementary file 4 — latex_source_files [file 42003_2023_4840_MOESM4_ESM.zip › Figures/Figure6.pdf]

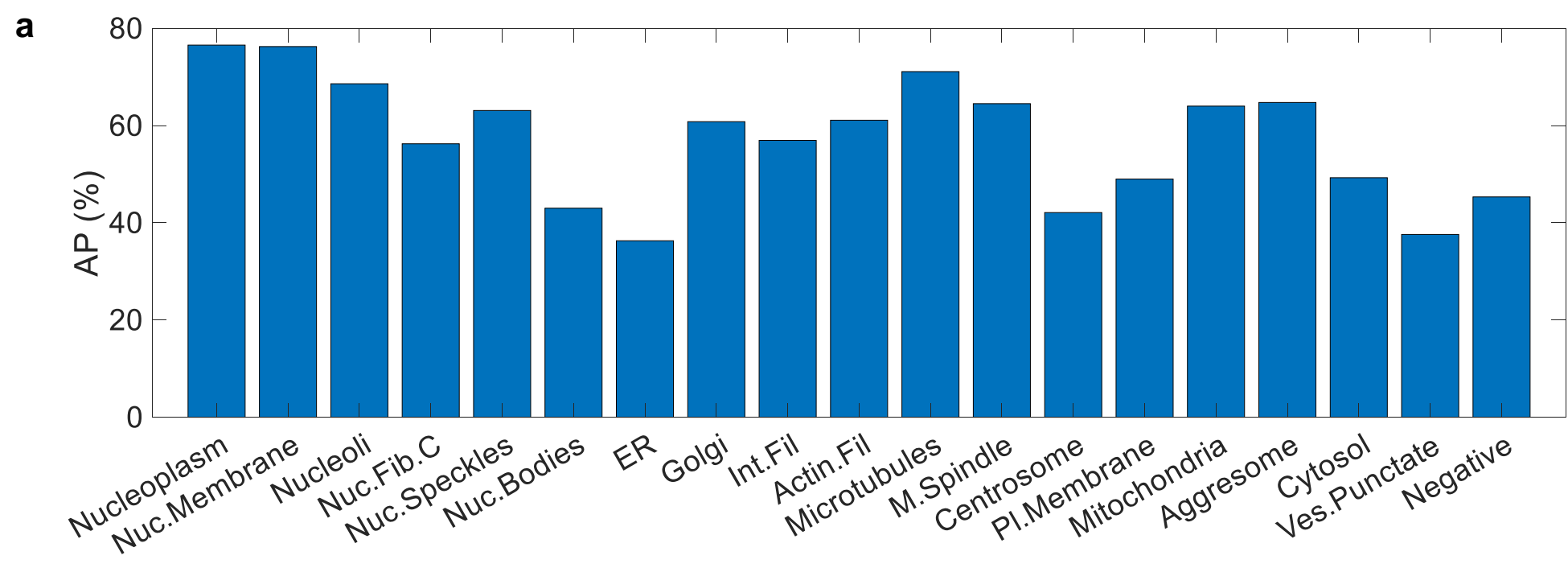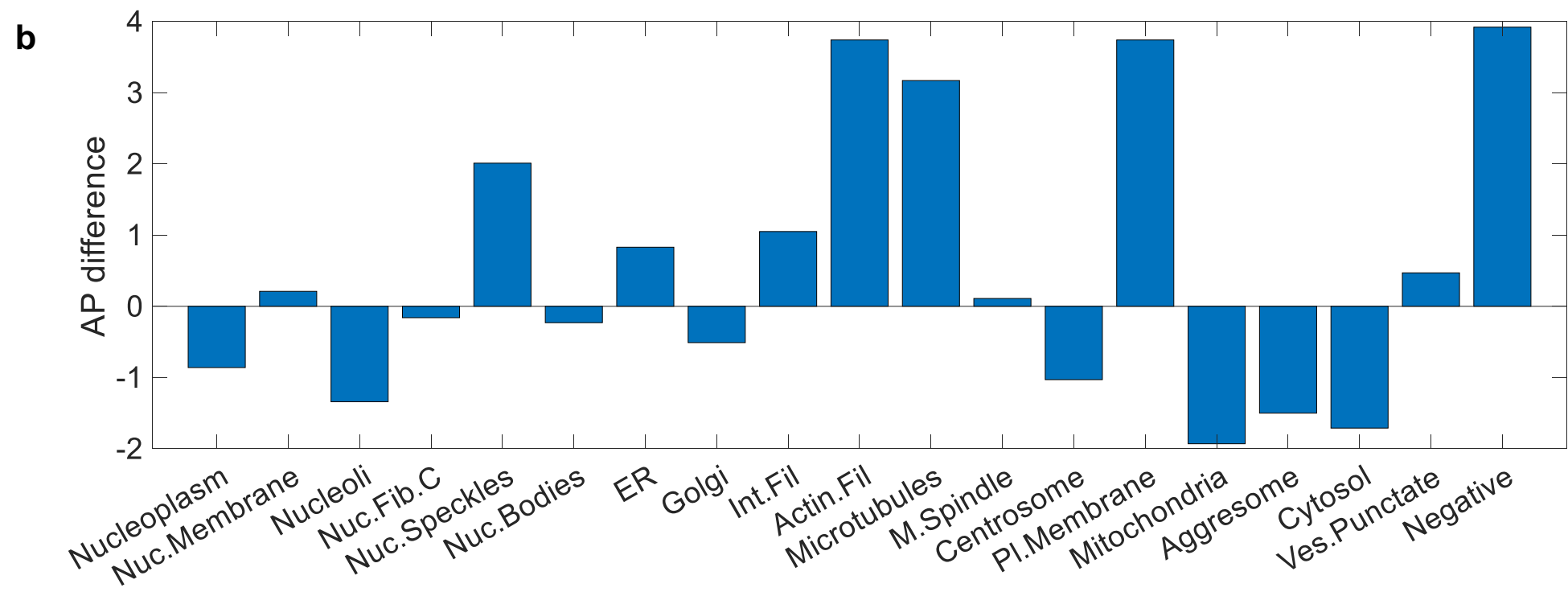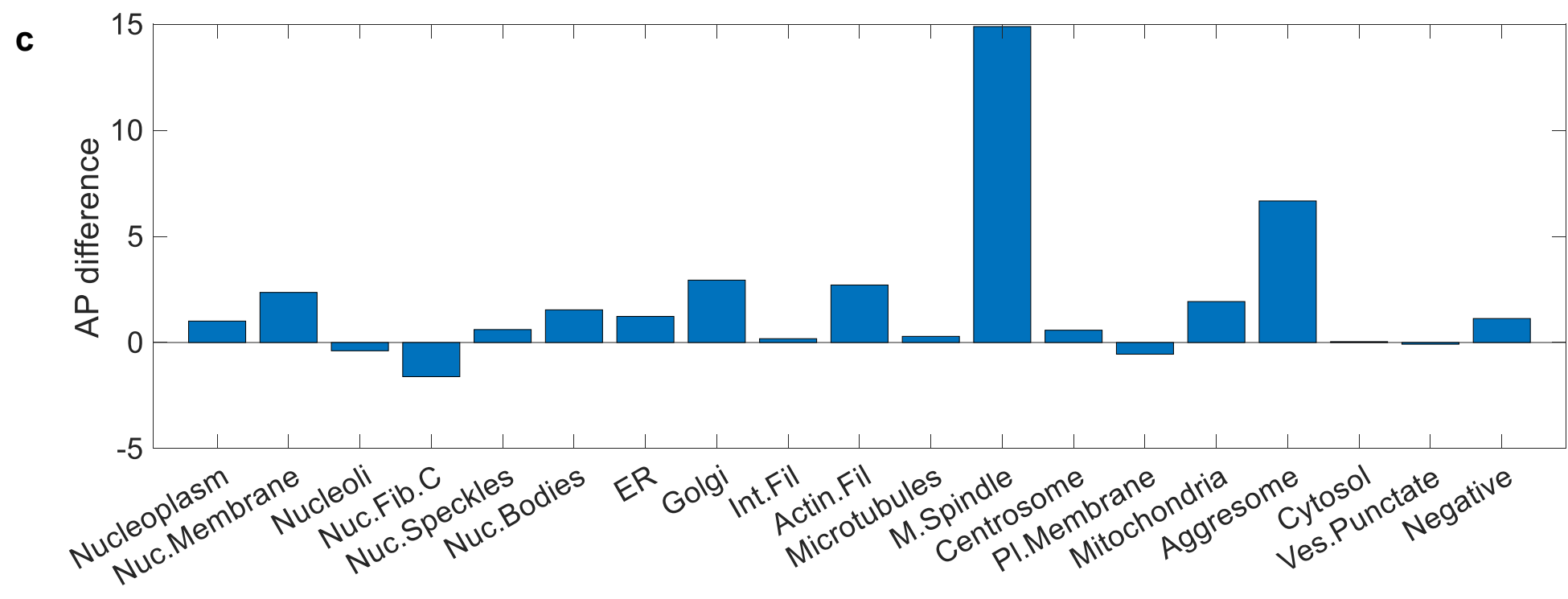

Supplement: Supplementary file 4 — latex_source_files [file 42003_2023_4840_MOESM4_ESM.zip › Figures/Figure7.pdf]

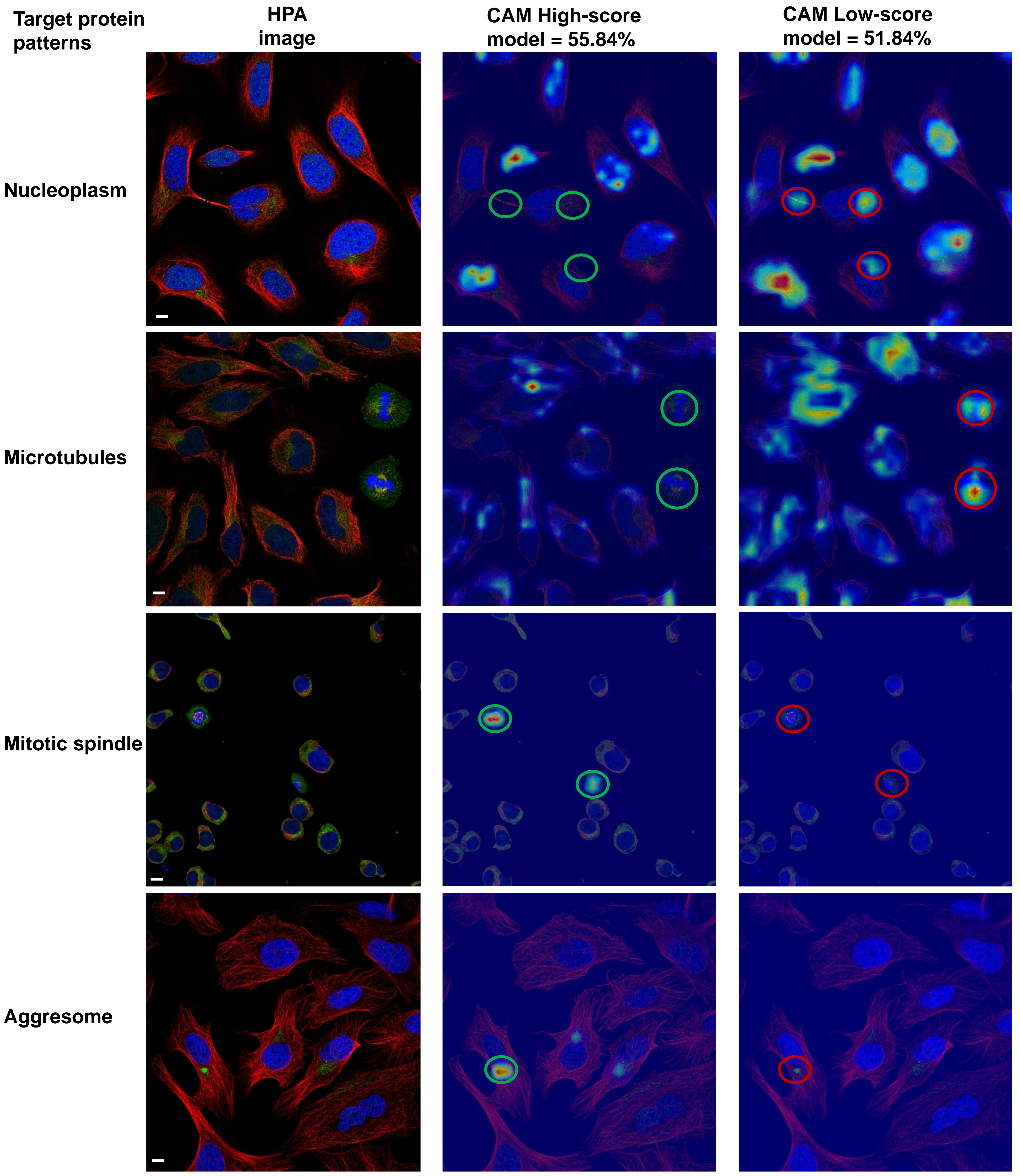

Supplement: Supplementary file 4 — latex_source_files [file 42003_2023_4840_MOESM4_ESM.zip › Figures/Figure8.pdf]
