# Supplementary material for: Single-cell subcellular protein localisation using novel ensembles of diverse deep architectures
Source: Commun Biol. 2023 May 5;6:489. doi: 10.1038/s42003-023-04840-z (PMC10163260; doi:10.1038/s42003-023-04840-z)
Supplement: Supplementary file 1 — Husain_Peer Review File [file 42003_2023_4840_MOESM1_ESM.pdf]

nature portfolio

Peer Review File

Single-cell Subcellular Protein Localisation Using Novel Ensembles of Diverse Deep Architectures

Open Access This file is licensed under a Creative Commons Attribution 4.0 International License, which permits use, sharing, adaptation, distribution and reproduction in any medium or format, as long as you give appropriate credit to the original author(s) and the source, provide a link to the Creative Commons license, and indicate if changes were made. In the cases where the authors are anonymous, such as is the case for the reports of anonymous peer reviewers, author attribution should be to 'Anonymous Referee' followed by a clear attribution to the source work. The images or other third party material in this file are included in the article's Creative Commons license, unless indicated otherwise in a credit line to the material. If material is not included in the article's Creative Commons license and your intended use is not permitted by statutory regulation or exceeds the permitted use, you will need to obtain permission directly from the copyright holder. To view a copy of this license, visit http://creativecommons.org/licenses/by/4.0/.

Reviewers' comments:

Reviewer #1 (Remarks to the Author):

The authors constructed a Hybrid subCellular Protein Localiser (HCPL) with weakly labelled IF image data to recognize protein subcellular patterns in single cells. The HCPL combined several novel deep neural networks and the experimental results showed that HCPL could achieve better performance than state-of-the-art methods. The authors emphasized the important role of reliable labels for training, and did a lot of work to improve the quality of the weak labels. In addition, the contribution of sub-models to HCPL had been analyzed. Currently, research about single-cell protein location is still in its infancy, the methods used in this paper provide a good reference.

1. The authors claimed that they conducted a series of ablation studies, but they showed only the comparison of sub-models before and after ensemble, and comparison with other Kaggle models, which were undescribed. Firstly, please give a brief description of the competition models, so that we could be aware of the innovative parts of the HCPL. Secondly, the modules used in HCPL, such as the hybrid fusion block for CLH and learnable Weibull activation pooling for CLA, are clearly important, so their ability should be studied through ablation experiments.
2. Some methods and chosen parameters used were not clearly described, such as the hand-crafted features used in the CLH, the weights used in the weighted binary cross-entropy loss, the threshold ρ, and the weights for image stream loss and cell stream loss.
3. The accuracy of the Visual Integrity Detector should be given.
4. It is not clear how the two VID modules in Visual Integrity Detector system were combined to get VI weights.
5. In page 6 ‘A class-wise comparison demonstrates that the CLH better represents rare classes (0.22% and 0.11%mAP...)’. mAP is the mean value of all classes. It seems unreasonable to use the mean value to show that the gain is brought by a single class. The detail of ‘class-wise comparison’ could be provided to strong the results.
6. According to the reference 11, there are 1776 images in the Kaggle test set, which was divided into a public test set with 559 images and a private test set with 1217, but those numbers in this paper are different.
7. What is the ‘HCL Classification Layer’ in Fig. 2b?
8. The y-axis in Fig.5e should be AP.
9. Would the code be publicly available?

Reviewer #2 (Remarks to the Author):

In this paper, the authors propose an ensemble of machine-learning models to identify protein localization at single-cell resolution. This work tackles the challenge of multilabel classification even though the given label information might be wrong. This work is based on the datasets provided by Human Protein Atlas (HPA) and a Kaggle competition hosted by HPA. The significance of this work lies in the fact that it outperforms the winning model of the Kaggle competition by 0.43%. However, all the models submitted at the competition were trained without knowing the private test data, whereas this work was conducted under the condition that all the private test data were revealed. Therefore, it is unfair to claim that this work "defines state-of-the-art" based on the 0.43% improvement. As the Kaggle leaderboard indicates, the difference of 0.43% is very small and any top models could have achieved this level of improvement if the private test data was revealed. Kaggle has split the test data into public and private particularly to prevent overfitting on the test data (https://www.kaggle.com/c/home-data-for-ml-course/overview/frequently-asked-questions) because this can inevitably happen consciously or subconsciously.

The manuscript is not well polished (see below), missing many details (see below), and overall overselling its novelty and performance with unconvincing data visualization (see below). Considering the code is not publicly available, little contribution can be made to the community of biological sciences.

• There is no statement about code availability in the manuscript even though the journal policy requires "For all studies using custom code that is deemed central to the conclusions, a statement must be included in the Methods section, under the heading "Code availability", indicating whether and how the code can be accessed, including any restrictions."
• The evaluation of models performed on Kaggle private leaderboard could not be accessed at https://www.kaggle.com/competitions/hpa-single-cell-image-classification/submissions
• Fig. 1 is too busy and hard to read/understand. Some information is redundant or not substantially related to the core of this work and should be omitted or moved to supplementary information. Other information is misleading or wrong.
• For example, Fig. 1a is not showing a "workflow" but is just listing some sample images. There is nothing that indicates flow. It shows "Cell culturing" but there is no description of cell culture in the text. What does the silhouette of a microplate mean? The illustration of the microscope is misleading. The microscope is an upright microscope, which cannot image cells cultured in dishes or well plates.
• In fig. 1b, the endoplasmic reticulum channel should be yellow only according to the figure caption but appears to have red color.
• The graphical illustration in fig. 1c hardly conveys anything. It can only make sense after I read the figure caption. It is a duplicated effort for the readers if the figure is intended to offer a big picture but requires readers to read texts to understand what the figure means. In addition, I still could not quite understand how the given images are related to "Poor segmentation."
• What are the "eight morphological features" used in Visual Integrity Detector (VID)?
• The manuscript excessively uses overselling words such as novel, highly performing without giving

evidence. For example, how Cell-level Actnet (CLA) is “highly performing”? Compare to what? How CLA is “novel” compared to the previous Actnet (ref. 19)?

• It seems that “rare classes are given more weight than frequent classes” but how? How were the weight values determined for each class?

• Fig. 5e shows “The performance comparison of HCPL and HPA challenge top 6 teams [11] for different label classes.” but does not mention if the bars for the HPA challenge top 6 teams are average values or median or something else. Also, why do the authors compare the performance of a single model with the combined values of 6 teams? I could understand if the authors compared the performance of HCPL with that of the top 1 team or the max values from the top 6 teams. Or just show the scores of all 6 teams. If the authors compared the performance of HCPL with the average values of the top 50 teams (which is provided by ref. 11), the superiority of HCPL would be even more obvious.

Response to comments from reviewers (21 January 2023)

Single-cell Subcellular Protein Localisation Using Novel Ensembles of Diverse Deep Architectures. Authors: Syed Sameed Husain, Eng-Jon Ong, Dmitry Minsky, Mikel Bober-Irizar, Amaia Irizar and Miroslaw Bober

We would like to thank the reviewers and the deputy editor for their time and detailed comments, which will help us improve the manuscript. We have addressed each comment of the reviewers.

Reviewer 1

Comment*: The authors constructed a Hybrid subCellular Protein Localiser (HCPL) with weakly labelled IF image data to recognize protein subcellular patterns in single cells. The HCPL combined several novel deep neural networks and the experimental results showed that HCPL could achieve better performance than state-of-the-art methods. The authors emphasized the important role of reliable labels for training and did a lot of work to improve the quality of the weak labels. In addition, the contribution of sub-models to HCPL had been analysed. Currently, research about single-cell protein location is still in its infancy, the methods used in this paper provide a good reference.*

Response: Many thanks for the positive comments on the HCPL impact and performance on subcellular protein localisation in single cells task.

Comment 1a: *The authors claimed that they conducted a series of ablation studies, but they showed only the comparison of sub-models before and after the ensemble, and comparison with other Kaggle models, which were undescribed. Firstly, please give a brief description of the competition models, so that we could be aware of the innovative parts of the HCPL.*

Response 1a: Below we provide a brief description of the competition models. We have added these details in the Supplementary section 1 of the manuscript.

The approaches used in the Kaggle competition [1] for classifying individual cells employed deep learning with two distinct models. The first is a Cell-level model (CLM), which takes segmented cells as input and outputs the predictions. An example of CLM is the Bag-of-Cells model [2], where the deep features from N cells in an image (bag) are extracted by a base CNN and aggregated using Mean pooling. The pooled tensor is forwarded to the learnable Attention pooling layer responsible for learning the weights for each cell representation belonging to a particular image: higher weighting is given to discriminative cells compared to the non-discriminative cells. The weighted representations are then pooled using the sum pooling function. Finally, the BCE loss is computed between the image label and image-level prediction. A more data-centric approach to developing CLM [3] involves re-labelling the cells using manual labelling and Meta Pseudo labelling algorithms. The CNN is then trained on refined data using Focal loss.

The disadvantage of CLM is inferior classification performance due to training using weak cell labels. To address this, Multi-head models (MHMs) were developed. The MHMs comprise an image head that models the entire population of cells and a cell head that classifies patterns in each cell in the image. An example of MHM is the Fair Cell Activation Network (FCAN) [4], an improved version of Puzzle-CAM [31], trained to jointly minimise multiple loss functions: reconstructing regularisation loss between the image and cells Class Activation Maps (CAMs), classification losses supervised by nineteen class labels and metric learning losses supervised by antibody labels. The Duo-branch CNN [5] is another example of MHM, where a random sample of N cells is first extracted from an image and then forwarded to a CNN. The last convolutional features from the CNN are aggregated and fed to an image head and a cell head. The fully connected (FC) layer of the cell head generates probabilities for each cell, while the FC layer of the image head outputs a single image probability. Finally, the predictions from two heads are passed to respective Binary Cross Entropy losses, and the final loss is the sum of losses from two heads. Another approach is the modified Puzzle-CAM [6] which consists of a Siamese network with reconstructing regularization loss that reduces the differences between the CAM of the original image and merged CAMs of image patches.

Comment 1b: *Secondly, the modules used in HCPL, such as the learnable Weibull activation pooling for CLA and hybrid fusion block for CLH, are clearly important, so their ability should be studied through ablation experiments.*

Response 1b: Thanks for pointing this out. We have added the additional ablation experimental results in the revised manuscript.

We performed experiments to highlight the importance of Weibull activation pooling for CLA and hybrid fusion block for CLH.

The Weibull activation pooling transforms convolutional layer values in an adaptive and non-linear manner: dampening the weaker responses and improving the discriminative power and robustness of stronger responses. Furthermore, Weibull pooling has the ability to equalise strong activations and hence prevents outliers from dominating the aggregated descriptor. We performed experiments to demonstrate that the Weibull pooling improves the mAP score by 2.82%, 2.53%, 2.11% and 1.95% compared to Global max pooling, Global average pooling, Region of interest pooling and Attention-based pooling.

The Hybrid fusion block for CLH effectively fuses information extracted by wavelet and scaling filters with learnt CNN features. The key idea is that instead of replacing the initial convolutional network stages with pre-defined filters, we enrich the learnt convolutional features with the outputs of the scattering transform. We design this fusion procedure to allow scattering features to guide the training process, i.e., enrich the data flow with representative pre-defined features available at the start of the training. As a result, the CLH benefits from well-defined and informative hand-crafted features, as well as from an adaptable and flexible deep learning-based approach. Experimental results show that the

Hybrid fusion blocks improve the performance by 1.58% mAP compared to the same DNN without fusion.

Comment 2: *Some methods and chosen parameters used were not clearly described, such as the hand-crafted features used in the CLH, the weights used in the weighted binary cross-entropy loss, the threshold ρ, and the weights for image stream loss and cell stream loss.*

Response 2: Thanks for pointing this out. We have added the following details in the revised manuscript.

<u>Hand-crafted features used in the CLH</u>

We employ scattering transform as the hand-crafted part of our hybrid network. It is formed by cascading wavelet transforms and modulus nonlinearity functions. It guarantees translation invariance and linear response to deformations by separating the information along with multiple scales and orientations.

<u>Weights used in the weighted binary cross-entropy loss</u>

The BCE weights for each class are computed as the inverse of the class frequency in the training dataset.

<u>Threshold ρ</u>

The value of threshold ρ is set to 0.32 for all models.

<u>Weights for image stream loss and cell stream loss</u>

In the first round of DSA training, the cell-level labels are weak, and we therefore intuitively assign a much lower weight to cell stream loss (L_2) compared to image stream loss (L_1). The final loss is computed as $L_f = 1 \times L_1 + 0.2 \times L_2$

In the second round, the DSA is trained on improved cell labels obtained from our CRA algorithm. Since we now have more trust in the cell-level labels the final loss is computed as $L_f = 1 \times L_1 + 1 \times L_2$. We have added these details in the Methods section.

Comment 3: *The accuracy of the Visual Integrity Detector should be given.*

Response 3: The accuracy of the VID is 92.05% on 2K cell images of the VID dataset. We have added the details in the revised manuscript.

Comment 4: *It is not clear how the two VID modules in the Visual Integrity Detector system were combined to get VI weights.*

Response 4: Thanks for pointing this out. We have added the details in the manuscript.

For training of the first VID module, we first compute the eight most representative features from each cell in the training dataset: bounding box height, width, aspect ratio, area, mask area, mask perimeter, the value of the largest dimension and a binary feature that is based

on the pixel intensity and the ratio of blue and green to the total number of pixels. The VID dataset contains 10K cells hand labelled as either 'good', i.e., most of a cell is visible or 'bad', i.e., a cell is severely occluded or a significant part not visible. We then train the XGBoost classifier on cell features using a fivefold cross-validation strategy. At inference time, each cell is passed to the XGBoost classifier to output the probability of the cell being bad (P_b). The VI weight W_1 is computed as $(1 - P_b)$.

The second module consists of a base EfficientNet-B2 with a fully connected layer to output predictions for four classes. The data to train the DNN is generated by randomly cropping out some area on the cell's border. The cells are classified into four classes based on the proportion of their cropped area to the original cell: less than 30% belongs to class 1, 30-50% belongs to class 2, 50-80% belongs to class 3, and 80-100% belongs to class 4. The network is trained using cross-entropy loss. During inference, the network is applied to each cell, and the weight W_2 is assigned a value based on the output class, with W_2 = 0.1 for class 1, W_2 = 0.5 for class 2, and W_2 = 1 for class 3 or 4.

The total weight W_v of VID is computed as the product of W_1 and W_2. Finally, the class probability of each cell is multiplied by W_v. The accuracy of the VID is 92.05% on 2K cell images of the VID dataset. The inclusion of the VID module mitigates false positive cell predictions and improves the system performance by 0.85% mAP.

Comment 5: *In page 6 'A class-wise comparison demonstrates that the CLH better represents rare classes (0.22% and 0.11%mAP...)'. mAP is the mean value of all classes. It seems unreasonable to use the mean value to show that the gain is brought by a single class. The detail of 'class-wise comparison' could be provided to strong the results.*

Response 5: Since only one class is involved it should be AP rather than mAP. We have amended this issue in the revised manuscript. Furthermore, the Figure below demonstrates the class-wise performance difference between Cell-Level Hybrid and Cell-Level Actnet networks. This Figure is also added to revised manuscript (Figure 3b).

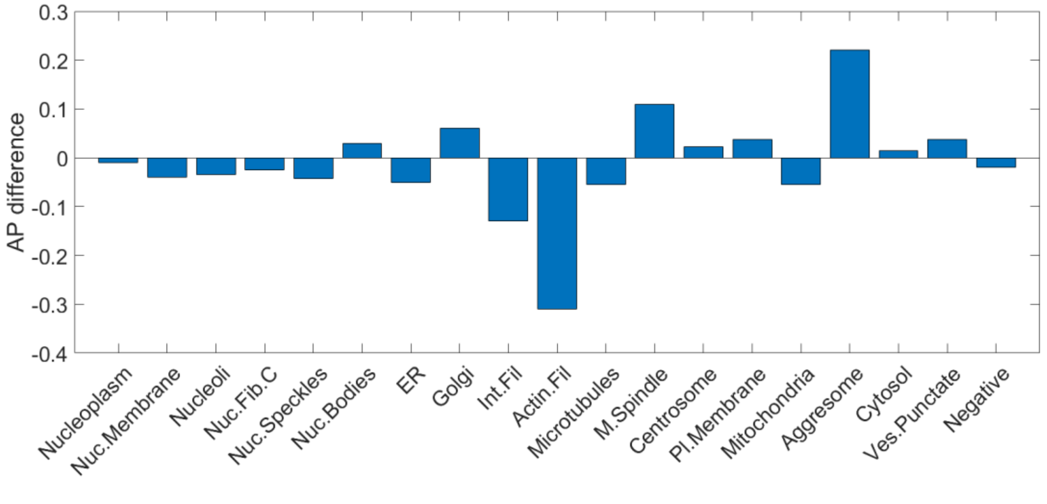

Figure 1: Class-wise performance difference between Cell-Level Hybrid and Cell-Level Actnet. Positive values indicate better performance for CLH.

Comment 6: *According to reference 11, there are 1776 images in the Kaggle test set, which was divided into a public test set with 559 images and a private test set with 1217, but those numbers in this paper are different.*

Response 6: Thanks for pointing this out. We have added the exact details of the dataset from [11] in our manuscript. The test dataset contains 1776 images. The test set was divided into a public test set with 31% of the images (n=559) and a private test set with 69% of the images (n=1,217), which has never been published.

Comment 7: *What is the 'HCL Classification Layer' in Fig. 2b?*

Response 7: Sorry about the error. This is amended in revised manuscript as FC classification layer.

Comment 8: *The y-axis in Fig.5e should be AP.*

Response 8: Yes, that is correct. We have amended it.

Comment 9: *Would the code be publicly available?*

Response 9: Yes, the authors will make the code publicly available. An appropriate "Code availability" statement is included in the Methods sections. In the interim, we make the code available to the reviewers (See code availability section).

Reviewer 2

Comment 1a*: In this paper, the authors propose an ensemble of machine-learning models to identify protein localization at single-cell resolution. This work tackles the challenge of multilabel classification even though the given label information might be wrong. This work is based on the datasets provided by Human Protein Atlas (HPA) and a Kaggle competition hosted by HPA. The significance of this work lies in the fact that it outperforms the winning model of the Kaggle competition by 0.43%. However, all the models submitted at the competition were trained without knowing the private test data, whereas this work was conducted under the condition that all the private test data were revealed. Therefore, it is unfair to claim that this work "defines state-of-the-art" based on the 0.43% improvement.*

Response 1a: We would like to clarify that the private test data is not available to anyone except competition organisers. Our models are trained using only the training dataset without any access to private test dataset and labels, and without external datasets. We used scores on the public test set to develop models, and report performance on the private test set. We have added this detail in the revised manuscript.

Comment 1b: *As the Kaggle leaderboard indicates, the difference of 0.43% is very small and any top models could have achieved this level of improvement if the private test data was revealed.*

Response 1b: We again want to emphasise the point that the private test set is not accessible to anybody except the organisers. Our HCPL system attains the single cell classification accuracy of 57.19% mAP. The best currently published result (56.67%, bestfitting [33]) was trained with protein-id or antibody-id information; such an approach is generally discouraged as it is known to generalise poorly on unseen cell lines or/and new staining markers. As mentioned by the competition organisers in [9], it was reported that gains achieved using this information might be due to the exploitation of data leakage between the training and testing examples via the antibody identifiers and cell-line information specific to the competition data.

Notably, the competition organiser, Emma Lundberg, specifically state that the antibody-id information cannot be disclosed. While antibody-id information can be used for better performance due to exploiting unintended hidden variables, it would not give the scientists better performance in discovering and understanding unknown proteins in laboratory research as this information is not always available (Human Protein Atlas - Single Cell Classification | Kaggle).

The evaluation results show that without using the antibody-id information, the performance of bestfitting is 55.54%. Importantly, no other team, including our HCPL, used antibody-id information during model training. When a fair comparison is performed, i.e., antibody-id data is not employed, HCPL (57.19%) demonstrates a gain of +1.65% and +1.86% over the first (55.54%) and second (55.33%) Kaggle teams. The gap is equivalent to the difference between the top nine solutions in the original competition.

We have added these details in the revised manuscript.

Comment 1c: *The manuscript is not well polished (see below), missing many details (see below), and overall overselling its novelty and performance with unconvincing data visualization (see below). Considering the code is not publicly available, little contribution can be made to the community of biological sciences.*

Response 1c: We thank the reviewer for pointing out imperfections which we have now addressed. We have responded to each comment and modified Figure 1 (see Response 3).

Regarding the code, we will make it publicly available. An appropriate "Code availability" statement is included in the Methods sections. In the interim, we make the code available to the reviewers (See code availability section).

We provide several new elements including new DNNs architectures (Dual-stream Actnet, VID and AI-trains-AI algorithm). Our work concentrates on the challenges that single-cell classification imposes and presents a systematic and in-depth solution, parts of which can be employed as stand-alone applications in a wider context (increasing the accuracy of existing labels and reducing the labour required for manual labelling tasks).

HCPL achieves a mAP of 57.19% in the single-cell classification of protein localisation patterns. HCPL can be used in numerous biological applications, especially where there is a lack of data annotations.

Comment 2: *There is no statement about code availability in the manuscript even though the journal policy requires "For all studies using custom code that is deemed central to the conclusions, a statement must be included in the Methods section, under the heading "Code availability", indicating whether and how the code can be accessed, including any restrictions."*

Response 2: Regarding the code, we will make it publicly available. An appropriate "Code availability" statement is included in the Methods sections. The Python source code for HCPL and model weights suitable for inference are available online via the project's web page at https://bit.ly/hpa-hcpl with the following access code: "TMWkA76Q".

Comment 3: *Fig. 1 is too busy and hard to read/understand. Some information is redundant or not substantially related to the core of this work and should be omitted or moved to supplementary information. Other information is misleading or wrong. For example, Fig. 1a is not showing a "workflow" but is just listing some sample images. There is nothing that indicates flow. It shows "Cell culturing" but there is no description of cell culture in the text. What does the silhouette of a microplate mean? The illustration of the microscope is misleading. The microscope is an upright microscope, which cannot image cells cultured in dishes or well plates. In fig. 1b, the endoplasmic reticulum channel should be yellow only according to the figure caption but appears to have red colour. The graphical illustration in fig. 1c hardly conveys anything. It can only make sense after I read the figure caption. It is a duplicated effort for the readers if the figure is intended to offer a big picture but requires readers to read texts to understand what the figure means. In addition, I still could not quite understand how the given images are related to "Poor segmentation"*

Response 3: We thank the reviewer for comments on Figure 1 which has now been modified substantially. Below we present the modified version.

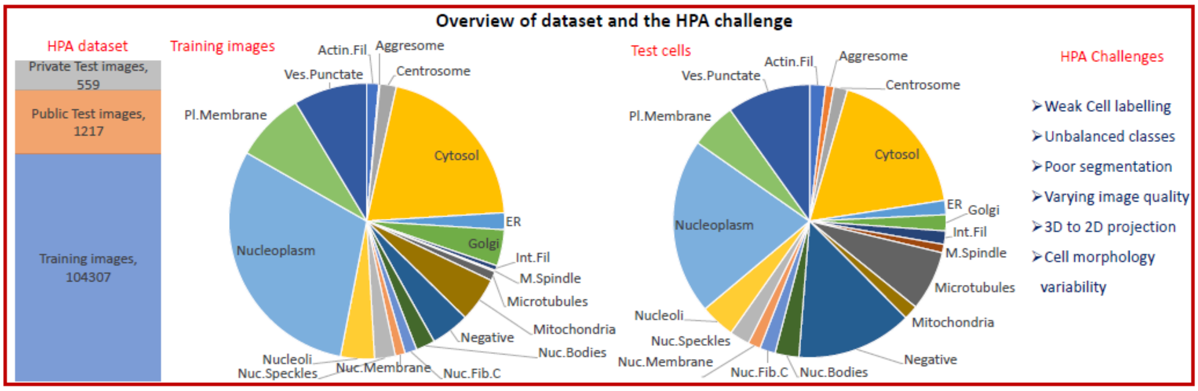

(a)

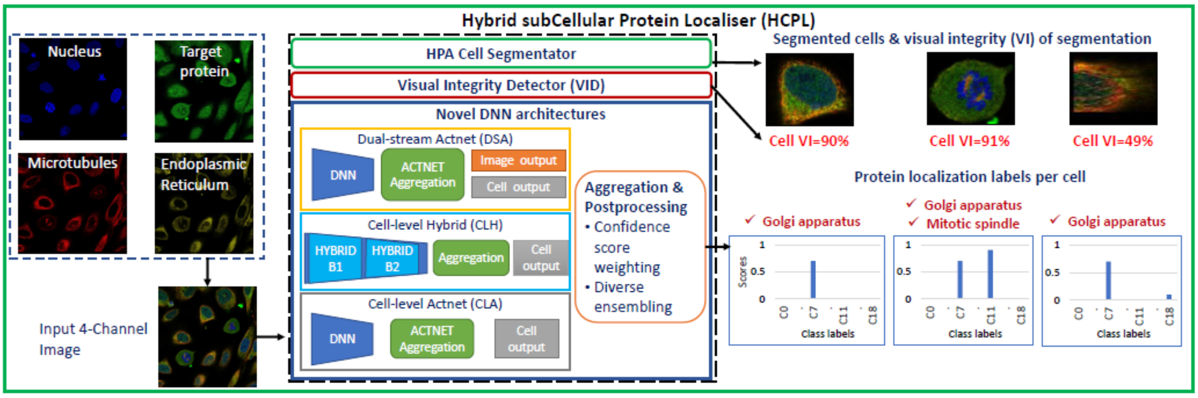

(b)

Figure 2: ***Overview of the HPA dataset and the proposed solution****.* ***a****, The HPA dataset is the largest collection of images depicting specific protein localisations at a subcellular level, acquired using immunofluorescence staining followed by confocal microscopy imaging. The training dataset consists of 104307 images and corresponding image-level labels. To evaluate the system's performance, the test set comprises 1776 images of 41597 single cells. The test set is divided into a public test set (559 images) and a private test set (1217 images). The pie charts illustrate the numerical proportion of images and cells per class in the training and test set. Developing ML models for protein localisation is challenging due to issues from weak labelling, prevalent multi-label classifications, 3D-2D projection ambiguities and severe class imbalance.* ***b****, Each HPA image is represented by four channels, the nucleus (blue), the protein of interest (green), microtubules (red) and the endoplasmic reticulum (yellow). Our HCPL system takes 4-channel images as input and outputs segmented cells, protein localisation labels with associated probabilities and the visual integrity scores for each cell. Experimental evaluation shows that HCPL achieves the classification performance of 57.19% mAP in single cell analysis (see evaluation and analysis in Figures 6 and 8).*

Comment 4*: What are the "eight morphological features" used in VID?*

Response 4: The eight features from each cell are: bounding box height, width, aspect ratio, area, mask area, mask perimeter, the value of the largest dimension and a binary feature that is based on the pixel intensity and the ratio of blue and green to the total number of pixels. The details are already described in the Methods section. We now have added these details in the main text of revised manuscript.

Comment 5*: The manuscript excessively uses overselling words such as novel, highly performing without giving evidence. How Cell-level Actnet (CLA) is "highly performing"? Compare to what? How CLA is "novel" compared to the previous Actnet (ref. 19)?*

Response 5: Thanks for your comment. We have revised our statements in the manuscript and provided a technical explanation on what algorithm advance allowed us to achieve the improvement.

We first highlight the importance of Weibull activation pooling and CLH hybrid fusion block. The Weibull activation pooling transforms convolutional layer values in an adaptive and non-linear manner: dampening the weaker responses and improving the discriminative power and robustness of stronger responses. Furthermore, Weibull pooling has the ability to equalise strong activations and hence prevents outliers from dominating the aggregated descriptor. We performed experiments to demonstrate that the Weibull pooling improves the mAP score by 2.82%, 2.53%, 2.11% and 1.95% compared to current Global max pooling, Global average pooling, Region of interest pooling and Attention-based pooling.

The Hybrid fusion block for CLH effectively fuses information extracted by wavelet and scaling filters with learnt CNN features. The key idea is that instead of replacing the initial convolutional network stages with pre-defined filters, we enrich the learnt convolutional features with the outputs of the scattering transform. We design this fusion procedure to allow scattering features to guide the training process, i.e., enrich the data flow with representative pre-defined features available at the start of the training. As a result, the CLH benefits from well-defined and informative hand-crafted features, as well as from an adaptable and flexible deep learning-based approach. Experimental results show that the Hybrid fusion blocks improve the performance by 1.58% mAP compared to the same DNN without fusion.

We then compare our architectures with the methods employed in the Kaggle (HPA Single Cell Classification) competition. We first compare the performances of single models without ensembling. From Figure 6b, we observe that the proposed DSA performs better than Kaggle multi-head models, including Fair Cell Activation Network (FCAN) [33], Duo-branch [24], modified Puzzle-CAM [34] [35], and PfNet [36]. This DSA improvement can be attributed to the use of Weibull activation pooling to aggregate deep features, correlation-dependent thresholding for the fusion of the two streams, along with training on improved labels from CRA.

Among cell-level architectures, Figure 6c demonstrates that our CLA achieves better classification performance (55.84%) than Cell model InceptionV3 [33], Cell model SwinT [33], Data-centric CNN [37], Gridify and Gapmask [38] and Bag-of-Cells [39]. The advantage of CLA is associated with the use of learnable and parametric activation pooling to aggregate discriminatory features. We have shown for the first time the benefits of Weibull-based aggregation for image classification tasks.

When ensemble-based approaches are considered, Our HCPL system attains the single cell classification accuracy of 57.19% mAP. The best currently published result (56.67%, bestfitting [33]) was trained with protein-id or antibody-id information. When a fair comparison is performed, i.e. antibody-id data is not employed, HCPL (57.19%) demonstrates a gain of +1.65% and +1.86% over the first (55.54%) and second (55.33%) Kaggle teams. The HCPL's better performance is predominantly due to the combination of its component parts, which includes utilising DNN architectures with wavelet filters and Weibull activation pooling to effectively handle significant cell variations, employing the CRA algorithm to produce enhanced cell labels, using the VID system to evaluate the visual quality of cells, and combining diverse deep architectures through correlation-based ensembling.

We have added the details in the revised manuscript.

Comment 6: *It seems that "rare classes are given more weight than frequent classes" but how? How were the weight values determined for each class?*

Response 6: The BCE weights for each class are computed as the inverse of the class frequency in the training dataset. We have added the details in the revised manuscript.

Comment 7: *Fig. 5e shows "The performance comparison of HCPL and HPA challenge top 6 teams [11] for different label classes." but does not mention if the bars for the HPA challenge top 6 teams are average values or median or something else. Also, why do the authors compare the performance of a single model with the combined values of 6 teams? I could understand if the authors compared the performance of HCPL with that of the top 1 team or the max values from the top 6 teams. Or just show the scores of all 6 teams. If the authors compared the performance of HCPL with the average values of the top 50 teams (which is provided by ref. 11), the superiority of HCPL would be even more obvious.*

Response 7: Thanks for the valuable comment. We have removed Figure 5e and added Figure 7 to show the class-wise performance difference between HCPL and Kaggle teams. Figure 3 below demonstrates the class-wise average precision (AP) difference between HCPL and Kaggle top team Bestfitting. It is important to note that the HCPL was trained without using antibody-id information compared to bestfitting. Nevertheless, HCPL achieves superior AP in classes: Nuclear speckles (2.01%), Actin filaments (3.74%), Microtubules (3.17%), Plasma membrane (3.74%) and Negative (3.92%). On the other hand, the Bestfitting method achieves better AP in classes: Nucleoli (1.34%), Mitochondria (1.93%), Aggresome (1.50%) and Cytosol (1.71%).
Figure 4 below demonstrates the class-wise AP difference between HCPL and the existing best performer without antibody-id information, red.ai (Kaggle 2nd place). We can observe that HCPL compares favourably across the vast majority of classes. We also observe a higher performance advantage of our system in challenging classes due to the synergetic operation of the individual system's components. For example, Cell-level Actnets help select the most discriminative features to distinguish actin filaments (+2.71%) that are visually very similar to intermediate filaments.

Similarly, CLH benefits from pre-defined filters that help extract valuable features from the mitotic spindle (+14.89%) and aggresome (+6.67%) despite the small number of training samples. These features of HCPL combined with VID help achieve an advantage in challenging classes over the red.ai system.

We have added these Figures in the revised manuscript.

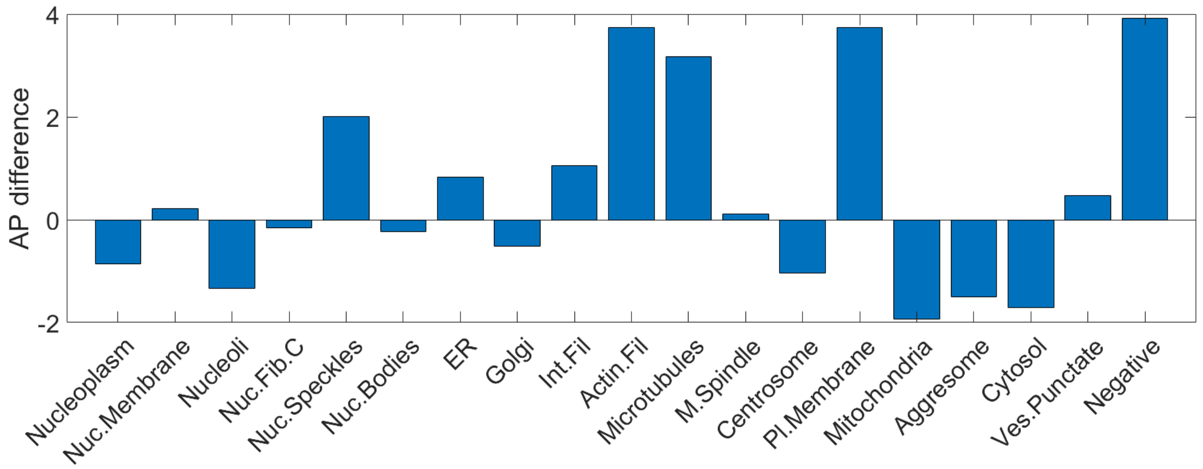

Figure 3: Class-wise performance difference between HCPL and Bestfitting (Kaggle top team). For each class we compute (HCPL AP - Bestfitting AP values). Positive values indicate better performance for HCPL.

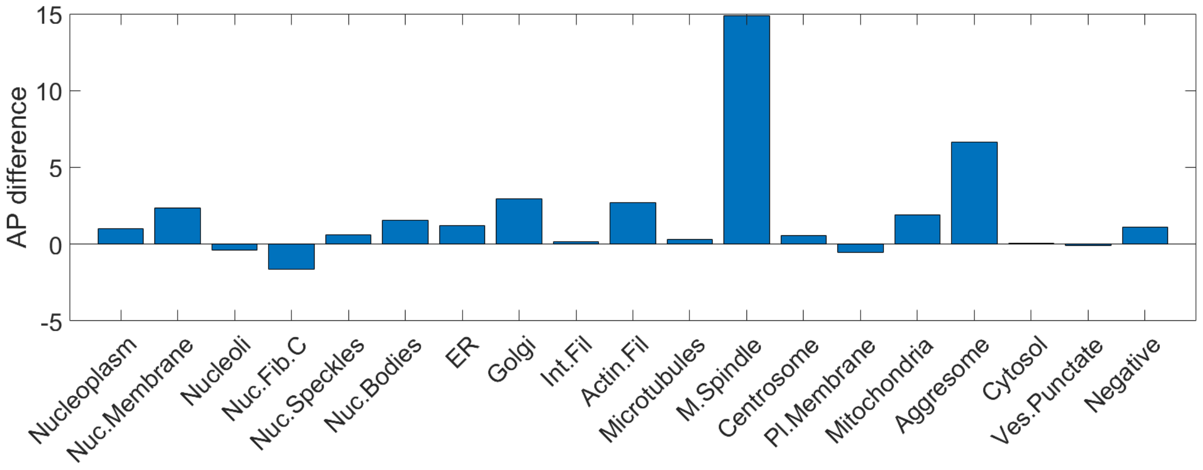

Figure 4: Class-wise performance difference between HCPL and Red.ai (Kaggle second team). Positive values indicate better performance for HCPL.

Reviewers' comments:

Reviewer #1 (Remarks to the Author):

The authors have addressed all my concerns.

Reviewer #2 (Remarks to the Author):

The manuscript has been improved substantially. There are a few things the authors might want to address to make the paper logically sound and scientifically accurate.

>Our models are trained using only the training dataset without any access to private test dataset and labels, and without external datasets. We used scores on the public test set to develop models, and report performance on the private test set.
>The test set is private and was not accessible to the authors; code submissions can be made for scoring through the Kaggle page.

I understand that the private test dataset is not accessible to the authors, but how many times did authors submit their code for scoring with the private dataset? In the real competition, the private scores are revealed only when the competition ends. After seeing the private scores, participants cannot modify their codes and resubmit. I wonder if the authors also only submitted the code once, and never changed their code after seeing their private scores. The authors should mention in the paper that if they have modified their code after seeing their private scores and resubmitted new code. If the authors did modify their code after seeing their private scores, it is equivalent to using the private dataset to develop models. I believe this is critical information to reveal if the authors want to provide a fair comparison.

>As mentioned by the competition organizers in [9], it was reported that gains achieved using this information might be due to the exploitation of data leakage between the training and testing examples via the antibody identifiers and cell-line information specific to the competition data.

Could the authors be more specific about which part of Ref. 9 indicates that “gains achieved using this information might be due to the exploitation of data leakage between the training and testing examples via the antibody identifiers and cell-line information specific to the competition data”? I could only find that there was data leakage in the early time of the competition but was fixed later. In addition, Ref. 9 does not describe anything about the competition [12] that the authors use in this paper. Instead, Ref. 11 describes the analysis of the competition [12] but I could not find anything about leakage.

>The best currently published result (56.67%, bestfitting [33]) was trained with protein-id or antibody-id information

>the competition organiser, Emma Lundberg, specifically state that the antibody-id information cannot be disclosed.

If “the antibody-id information cannot be disclosed”, how did bestfitting obtain antibody-id information? Did bestfitting use external data where antibody-id information was available? It is logically confusing, and the authors should make it clear.

>The evaluation results show that without using the antibody-id information, the performance of bestfitting is 55.54%.

How did the authors obtain 55.54%? If there is a reference, the authors should indicate it in the paper. If the authors retrained the winning model without using antibody-id information, the authors should provide more information about how the winning model or the training was modified.

>Notably, the competition organisers specifically state that the antibody-id information cannot be disclosed. While antibody-id information can be used for better performance due to exploiting unintended hidden variables, it would not give the scientists better performance in discovering and understanding unknown proteins in laboratory research as this information is not always available [40].

Here I quote the statement from the competition organizer:

It's important to keep in mind that we do this as part of discovering and understanding unknown proteins in laboratory research. While the protein information could potentially be used as input to better performance in this challenge, it would not give us better performance in the instances where we are hoping to be able to use the models from this challenge as the information may not always be available.

First of all, protein-id and antibody-id are not exactly the same information. Each type of protein is labeled by one or multiple antibodies in the HPA dataset but the expression in fluorescence images can vary among different antibodies and different cell types.
Second of all, the organizer did not disclose protein information because they want to discover and understand “unknown proteins.” They can still use known antibodies to probe unknown proteins. In fact, a lot of screening methods use known antibodies to investigate unknown proteins.
There is definitely an overlap between protein-id and antibody-id information but also not exactly the same. Therefore, I would advise the authors to be careful and unambiguous in the development of their statement, especially considering the majority of the readers are biologists.

It is hard to distinguish between fluorescence images and CAM when there are overlaid in Fig. 8(a). Could the authors separate them like Fig. 3 in Ref. 11?

Response to comments from reviewers (23 February 2023)

Single-cell Subcellular Protein Localisation Using Novel Ensembles of Diverse Deep Architectures. Authors: Syed Sameed Husain, Eng-Jon Ong, Dmitry Minsky, Mikel Bober-Irizar, Amaia Irizar and Miroslaw Bober

We would like to thank the reviewers and the deputy editor for their time and detailed comments, which helped us improve the manuscript. We have addressed each comment of the reviewers.

Reviewer 2

Comment 1: "Our models are trained using only the training dataset without any access to private test dataset and labels, and without external datasets. We used scores on the public test set to develop models, and report performance on the private test set. The test set is private and was not accessible to the authors; code submissions can be made for scoring through the Kaggle page."
I understand that the private test dataset is not accessible to the authors, but how many times did authors submit their code for scoring with the private dataset? In the real competition, the private scores are revealed only when the competition ends. After seeing the private scores, participants cannot modify their codes and resubmit. I wonder if the authors also only submitted the code once, and never changed their code after seeing their private scores. The authors should mention in the paper that if they have modified their code after seeing their private scores and resubmitted new code. If the authors did modify their code after seeing their private scores, it is equivalent to using the private dataset to develop models. I believe this is critical information to reveal if the authors want to provide a fair comparison.

Response 1: As per reviewer's request, we added the following explanation to the manuscript (line numbers 458-469):
A limitation of this comparison is that existing solutions were designed during the four-month Kaggle competition, with feedback based on public leaderboard scores, while we developed HCPL after the competition. While we had no access to test data, submitting code after the competition showed scores on public and private leaderboards. For a fair comparison, we only used the public leaderboard to develop our algorithm and avoided any optimisation based on the private leaderboard. Once we finalised the architecture based on the public leaderboard, we reported the performance on the private leaderboard.

Comment 2: "As mentioned by the competition organisers in [9], it was reported that gains achieved using this information might be due to the exploitation of data leakage between the training and testing examples via the antibody identifiers and cell-line information specific to the competition data."
Could the authors be more specific about which part of Ref. 9 indicates that "gains achieved using this information might be due to the exploitation of data leakage between the training and testing examples via the antibody identifiers and cell-line information specific to the competition data"? I could only find that there was data leakage in the early time of the competition but was fixed later. In addition, Ref. 9 does not describe anything about the competition [12] that the authors use in this paper. Instead, Ref. 11 describes the analysis of the competition [12] but I could not find anything about leakage.

Response 2: In [9, page 1259], it was stated that the metric learning model trained using antibody-id mainly gained performance by pairing images in the test set to those acquired from the same sample in the training set. With the help of CAMs, it was found that the visual attention pattern varied from protein to protein and often focused on biologically meaningless features. Presumably, the metric learning model works by exploiting 'batch effects' in the dataset through so-called 'hidden variables', a pitfall in machine learning.
In addition in [9, page 1260], it was stated that despite the superior performance of deep learning methods, there are limitations including hallucination and generalisation problems. It has also been reported that machine learning algorithms can easily pick up unintentional variations (for example, in biomedical image classification). The metric learning model trained with antibody-id used these unintended hidden variables (for example, background noise) to match the test images with the training set and boost the performance.
Furthermore, in [9, page 1262], it states that a compromise may be to restrict the set of information allowed for

training, such as only location labels from HPAv18 to avoid finding weak correlations with extra information such as antibody identifiers.
The antibody-ids were excluded from the competition datasets by the organisers because their use in training presents three issues: (1) the model may use irrelevant hidden variables (for example, background noise) or focus on biologically meaningless features to improve the model performance, (2) the gains achieved might be due to the exploitation of batch effects and statistical correlations between the training and test sets, and (3) the model trained using antibody-id may generalise poorly to unseen cell lines, new antibody-ids, and unknown proteins.
The data leakage in the early time of the competition [9] that was fixed later was of a different nature to the data leakage referred to in the manuscript. That problem was eliminated by removing images common to training and testing datasets. However, the possibility of the introduction of other biases, such as leakage due to antibody-id use or cell lines use, remained.
We have modified the wording in the manuscript (line numbers 489-507).

Comment 3: "The best currently published result (56.67%, bestfitting [33]) was trained with protein-id or antibody-id information. The competition organiser, Emma Lundberg, specifically states that the antibody-id information cannot be disclosed."
If the antibody-id information cannot be disclosed, how did bestfitting obtain antibody-id information? Did bestfitting use external data where antibody-id information was available? It is logically confusing, and the authors should make it clear.

Response 3: The antibody-id information was not provided by the competition organisers. Bestfitting obtained this information by scraping the Human Protein Atlas website and applying data mining algorithms. The details about this process is provided by bestfitting in Kaggle discussion [33].
We have added these details to the manuscript (line numbers 493-495).

Comment 4: "The evaluation results show that without using the antibody-id information, the performance of bestfitting is 55.54%." How did the authors obtain 55.54%? If there is a reference, the authors should indicate it in the paper. If the authors retrained the winning model without using antibody-id information, the authors should provide more information about how the winning model or the training was modified.

Response 4: We followed the bestfitting solution code (https://github.com/topics/hpa-challenge-2021) and the algorithm description [11, 33] to retrain the models without using the antibody-id information. The base model for the bestfitting solution, Fair Cell Activation Network (FCAN), was trained to jointly minimise three loss functions: reconstructing regularisation loss between the image and cells Class Activation Maps (CAMs), classification losses supervised by nineteen class labels and metric learning losses supervised by antibody-ids. We removed the metric learning loss layer and retrained the FCAN using the original hyper-parameters. Following this, we used the original post-processing algorithm and an ensemble of FCANs and cell-level models to obtain the final solution.
The details are added in the manuscript (line numbers 519-522 and Methods section lines 809-826).

Comment 5: "Notably, the competition organisers specifically state that the antibody-id information cannot be disclosed. While antibody-id information can be used for better performance due to exploiting unintended hidden variables, it would not give the scientists better performance in discovering and understanding unknown proteins in laboratory research as this information is not always available [40]."
Here I quote the statement from the competition organiser: It's important to keep in mind that we do this as part of discovering and understanding unknown proteins in laboratory research. While the protein information could potentially be used as input to better performance in this challenge, it would not give us better performance in the instances where we are hoping to be able to use the models from this challenge as the information may not always be available.
First of all, protein-id and antibody-id are not exactly the same information. Each type of protein is labelled by one or multiple antibodies in the HPA dataset but the expression in fluorescence images can vary among different antibodies and different cell types.
Second of all, the organiser did not disclose protein information because they want to discover and understand "unknown proteins." They can still use known antibodies to probe unknown proteins. In fact, a lot of screening methods use known antibodies to investigate unknown proteins. There is definitely an overlap between protein-id and antibody-

id information but also not exactly the same. Therefore, I would advise the authors to be careful and unambiguous in the development of their statement, especially considering the majority of the readers are biologists.

Reponse 5: We agree that protein-id and antibody-id are not exactly the same. However, they are directly linked - as the reviewer correctly notes, "each type of protein is labelled by one or multiple antibodies, but the expression in fluorescence images can vary among different antibodies and different cell types." While known antibodies can be used to probe unknown proteins, our and the organiser's concern relates to the potential bias that the use of such additional information (protein-id/antibody-id and cell lines) can introduce. Specifically, all machine learning (ML) methods build a probabilistic model that captures the statistics of the training datasets. The concern is that certain undesired statistics, such as linking prior probabilities of protein/antibody ids or cell lines to cellular locations, may not be preserved for the new, unknown proteins or even for known proteins that are expressed in different cell lines. This may introduce a bias that is difficult to detect or control.
Based on reference [40] and in the context of the discussions and conclusions from the previous competition (where organisers used specifically the terms protein/antibody and cell line together [9, page 1262]), we believe the organisers intended to exclude both protein and antibody id from training for both competitions). In fact, competition organisers suggested limiting the set of information allowed for training to protein location labels only (as supplied with the datasets). This prevents models from exploiting spurious correlations between the predicted protein localisation labels and additional information such as antibody-ids [9], protein-ids [42] and cell lines [43], helping to ensure that the cell localisation predictions are unbiased and generalise well.
We agree with the reviewer that the distinction between the antibody and protein id and their impact on ML task was not clear and therefore we modified the text to address this (line numbers 508-516).

Comment 6: It is hard to distinguish between fluorescence images and CAM when there are overlaid in Fig. 8(a). Could the authors separate them like Fig. 3 in Ref. 11

Response 6: Thanks for your comment. We have modified Figure 8(a). The Figure below (Figure 8 in the revised manuscript) now displays the original HPA images, CAMs of the high-scoring CLA trained on CRA improved labels, and a low-scoring CLA trained on original weak labels. We have also introduced a new Figure 9 in the revised manuscript which displays the Visualisation of the deep features for DSA and CLH DNNs from the UMAP dimensionality reduction method which was previously shown in Figure 8(b).

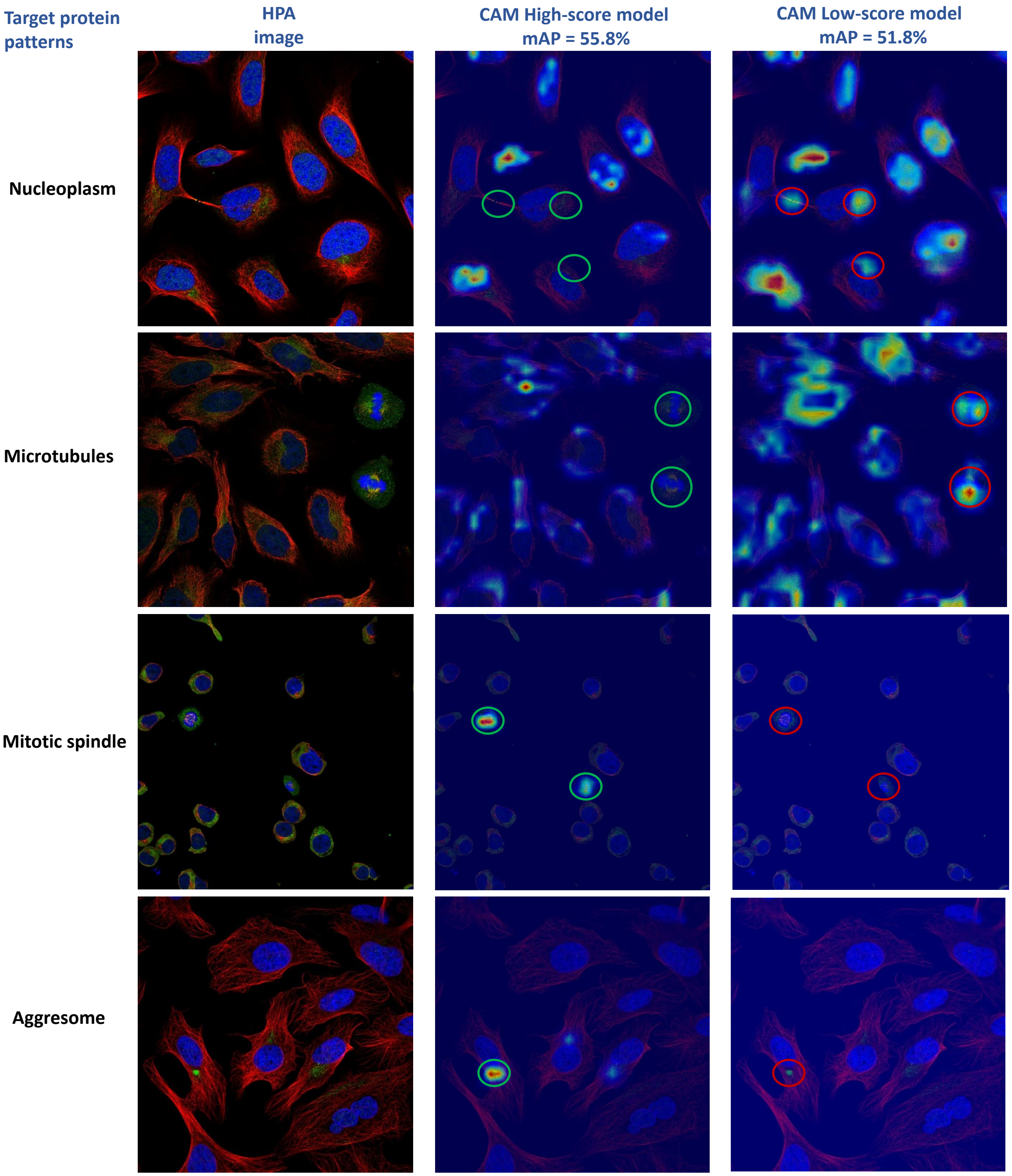

Figure 1: CAMs of the high-scoring CLA trained on CRA improved labels, and a low-scoring CLA trained on original weak labels. (1) The CAMs for the nucleoplasm staining demonstrate biologically relevant attention for the high-scoring model only as shown by green circles. (2) The microtubule staining overlaps well with the CAM for the high-scoring model. In contrast, the low-scoring model uses visual features of the mitotic spindle class to assign a high probability for the microtubule class, as indicated by red circles. (3) For the mitotic spindle staining patterns, CAMs for the high-scoring CLA highlight relevant cellular regions indicated by green circles. (4) The red circle shows where the aggresome staining pattern is not captured by the low-scoring DNN, whereas the green circle depicts the high-scoring model correctly localises it.

REVIEWERS' COMMENTS:

Reviewer #2 (Remarks to the Author):

The authors have addressed all my concerns.
